# Rhythmic Components in Extracranial Brain Signals Reveal Multifaceted Task Modulation of Overlapping Neuronal Activity

**DOI:** 10.1371/journal.pone.0154881

**Published:** 2016-06-23

**Authors:** Roemer van der Meij, Freek van Ede, Eric Maris

**Affiliations:** 1 Radboud University Nijmegen, Donders Institute for Brain Cognition and Behaviour, Montessorilaan 3, 6525 HR, Nijmegen, The Netherlands; 2 University of California San Diego, Department of Cognitive Science, 9500 Gilman Drive, La Jolla, California, 92093, United States of America; 3 Oxford Centre for Human Brain Activity, Department of Psychiatry, University of Oxford, OX3 7JX, Oxford, United Kingdom; Georgia State University, UNITED STATES

## Abstract

Oscillatory neuronal activity is implicated in many cognitive functions, and its phase coupling between sensors may reflect networks of communicating neuronal populations. Oscillatory activity is often studied using extracranial recordings and compared between experimental conditions. This is challenging, because there is overlap between sensor-level activity generated by different sources, and this can obscure differential experimental modulations of these sources. Additionally, in extracranial data, sensor-level phase coupling not only reflects communicating populations, but can also be generated by a current dipole, whose sensor-level phase coupling does not reflect source-level interactions. We present a novel method, which is capable of separating and characterizing sources on the basis of their phase coupling patterns as a function of space, frequency and time (trials). Importantly, this method depends on a plausible model of a neurobiological rhythm. We present this model and an accompanying analysis pipeline. Next, we demonstrate our approach, using magnetoencephalographic (MEG) recordings during a cued tactile detection task as a case study. We show that the extracted components have overlapping spatial maps and frequency content, which are difficult to resolve using conventional pairwise measures. Because our decomposition also provides trial loadings, components can be readily contrasted between experimental conditions. Strikingly, we observed heterogeneity in alpha and beta sources with respect to whether their activity was suppressed or enhanced as a function of attention and performance, and this happened both in task relevant and irrelevant regions. This heterogeneity contrasts with the common view that alpha and beta amplitude over sensory areas are always negatively related to attention and performance.

## Introduction

Neuronal signals contain oscillations at many frequencies [1], and these have been shown to be implicated in many cognitive functions (for a review, see [2]). It is commonly thought that oscillations reflect fluctuations of neuronal excitability [3], whose phase coupling may be used for the dynamic communication between neuronal populations [4, 5]. Accordingly, neuronal oscillations are of interest to a large scientific community, and phase coupling is a core component in many interpretations of experimental modulations of neuronal oscillations. A large share of this community studies oscillatory activity on the basis of extracranial recordings, such as electroencephalography (EEG) and magnetoencephalography (MEG).

### Challenges in the study of oscillatory neuronal activity in extracranial recordings

Investigating oscillatory activity in extracranial recordings (EEG/MEG) is challenging for several reasons. First, interpreting sensor-level activity is strongly hindered by the fact that the underlying sources generate overlapping sensor-level spatial patterns. This is especially problematic when neuronal activity is investigated in the context of a task, and the question of interest is whether activity differs as a function of experimental variables or behavior. This typically involves an analysis of some measure of neuronal activity (e.g. power, coherence) and an independent variable (e.g. conditions, reaction times) at every sensor or sensor-pair. This is suboptimal for two reasons. First, quantifying the reliability of an effect requires a statistical evaluation for each of the sensors (sensor-pairs), which is problematic without a priori hypotheses about where the effect will occur. Second, when sensor-level representations of neuronal sources overlap due to volume conduction or field spread, then only the sum of their activities can be related to the independent variable. As such, it may appear that sensor-level activity is either suppressed or enhanced by an experimental manipulation, whereas different underlying sources are each modulated differently (e.g. with some sources being suppressed, while others are enhanced, as we will show later).

Investigating neuronal activity in extracranial brain signals is further hampered by the fact that sensor-level phase coupling not only reflects communicating neuronal populations, but can also reflect activity that is best described by a current dipole, which is produced by a point source. Especially when the distance between sources and sensors is large, as for EEG/MEG, it is likely to observe sensor-level phase coupling that can be described by a current dipole.

One way to overcome these challenges is by methods that extract patterns of activity which are more informative of the underlying source activity. In this paper, we present a method that achieves this. It separates the activity patterns of sources whose spatial and spectral profiles strongly overlap, and allows for investigating their individual task modulations. Additionally, it allows for distinguishing between point sources, whose sensor-level phase coupling does not reveal interactions between neuronal subpopulations, and other sources, for which this *is* the case.

There are existing methods whose objective is also to separate activity from (overlapping) sources, and we will now briefly review these methods. Because we focus on oscillatory activity, we only consider methods for the frequency-domain representation of the signals (i.e., Fourier coefficients). Afterwards, we highlight three ways in which our method can improve upon existing methods.

### Existing methods for separating overlapping sources

It is useful to distinguish between methods that only operate on the amplitude of Fourier coefficients, and methods that also take their phase into account. Considering the first, we must further distinguish between methods that can only be applied to amplitudes in a single frequency band (also denoted as *frequency*), and methods that can be applied to amplitudes at multiple frequencies simultaneously. Considering the former, the best-known methods are Independent and Principal Component Analysis (ICA, PCA) [6, 7–10]. With these methods, one can describe the structure in a matrix of frequency-specific amplitude time-series collected at multiple sensors. More specifically, they decompose this matrix in a number of components with a fixed (time-invariant) spatial pattern and a fixed (spatially invariant) amplitude time course. Importantly, the components are identified using statistical constraints (orthogonality, maximum variance, statistical independence), which do not necessarily follow the neurophysiology of the phenomena under investigation.

Next, we consider a method that can be applied to Fourier amplitudes at multiple frequencies simultaneously: PARAllel FACtor analysis (PARAFAC) [11, 12–14]. PARAFAC operates on 3-way arrays of sensors-by-frequencies-by-time-points. It decomposes these arrays into components with a fixed (frequency- and time-invariant) spatial map, a fixed (space- and time-invariant) frequency profile, and a fixed (space- and frequency-invariant) amplitude time course. Crucially, these components are identified without additional statistical constraints.

Some methods also take the phase of Fourier coefficients into account, and this is advantageous for two reasons. First, it allows for inferring important aspects of the source configuration producing the measured neural activity [15, 16], and second, it provides additional information allowing for the separation of overlapping sources. Two such methods have been proposed for extracting patterns from arrays of Fourier coefficients involving a spatial, a spectral and a temporal dimension: complex-valued ICA [17, 18] and complex-valued PARAFAC [19]. Both methods have not yet been widely applied to neuroscience data. As for the real-valued case, different algorithms exist for complex-valued ICA, and they all identify components using a statistical constraint. Complex-valued PARAFAC does not require a statistical constraint, but instead assumes that the components are characterized by a fixed spatial map, a fixed frequency profile, and a fixed time course. From a neurobiological perspective, an important limitation of complex-valued PARAFAC is that a component’s complex-valued sensor loadings describe the between-sensor phase relations by a single set of phases. This results in between-sensor phase relations that are constant over frequencies. This is useful to describe, for instance, single oscillating point sources (because these induce frequency-invariant between-sensor phase relations equal to zero), but not for distributed source configurations whose phase relations vary over frequency. Shifted CANDECOMP/PARAFAC (Shifted CP) is an improvement upon complex-valued PARAFAC [20], but it can only describe phase relations induced by a small number of sources with time-shifted activity time courses.

### Three key properties of our proposed approach for analyzing oscillatory neuronal activity

In this paper, we present a novel approach for analyzing oscillatory neuronal activity, which uses a model-based method that separates and characterizes sources by their patterns of between-sensor phase coupling. It is a decomposition method that provides a parsimonious description of the structure in oscillatory neuronal activity. The method was first presented in [21]. Unlike existing decomposition methods, it uses a plausible model of a neurobiological rhythm: a spatially distributed oscillation with energy in a range of frequencies and involving between-sensor phase relations that can vary over frequencies. Because the model is formulated for rhythmic neuronal activity, we denote the extracted patterns as rhythmic components. These rhythmic components describe the sources that produce the sensor-level measurements.

Analyzing oscillatory activity using rhythmic components can improve upon existing approaches because of three key properties. First, it allows for a separation of sources with overlapping spatial and spectral patterns, and therefore can reveal sources which are difficult to isolate in conventional pairwise analyses. Secondly, the strength of a component is quantified for each trial by a single number (denoted as a *trial loading*). These trial loadings allow for a straightforward way of investigating task modulations of oscillatory activity at the level of the extracted components. Thirdly, identifying rhythmic components is a first step in the analysis of phase-coupled oscillatory networks, because they provide a parsimonious description of the interacting neuronal populations that have produced the pattern of phase coupling at the sensor-level.

## Materials and Methods

### Model and Parameter estimation

#### SPACE: A model-based method for characterizing between-sensor phase coupling by rhythmic components

Electrophysiological recordings reflect neuronal oscillations and the phase of these oscillations is often consistent between recording sites (sensors). We proposed a decomposition method based on a model that parsimoniously describes patterns of between-sensor phase coupling by rhythmic components. Importantly, this model is a neurobiologically inspired source model, and therefore describes patterns of between-sensor phase coupling in a way that is informative for this community [21]. This model-based method is denoted as SPACE (Spatially distributed PhAse Coupling Extraction).

To extract components, we start with electrophysiological measurements *V*_*jl*_(*t*) (potential differences or magnetic field strength) measured over time *t*, obtained from sensor *j* (total number *J*) and trial *l* (total number *L*). Oscillatory activity in these recordings is described by Fourier coefficients, which we obtain from a spectral analysis involving multitapering (e.g. Welch [22] or Slepian [23] tapering; multitapering is optimal because it provides control over the frequency resolution, but it is not required for our method). The obtained Fourier coefficients *X*_*jklm*_ describe the average amplitude and the phase of oscillations in each tapered part *m* of trial *l*, at frequency *k*, and at sensor *j*. We then compute the frequency- and trial-specific cross-spectral density (CSD) matrices. These are obtained from the cross products of the sensor-by-taper (*J* × *M*) matrices of Fourier coefficients *X*_*kl*_, as the average over tapers $\left(X_{kl}\cdot X_{kl}^{*}\right)/M$ (* denotes the complex conjugate transpose). A CSD reflects both the power in the different sensors and the between-sensor phase consistency. The model underlying our method (presented below) describes the systematic variability of amplitudes and phases of $X_{kl}\cdot X_{kl}^{*}$ by multiple components, each consisting of four parameter sets. The first of these, the frequency profile describes which frequencies are involved in between-sensor phase coupling. The second parameter set, the spatial amplitude map describes which sensors are phase-coupled, at the frequencies in the frequency profile. The third parameter set, the spatial phase maps describe, per frequency, the consistent between-sensor phase relations. Finally, the fourth set, the trial profile quantifies how strongly each component is present in each trial, and can be viewed as a measure of activity of the neuronal source that is reflected by this component. The spatial amplitude map and the spatial phase maps describe phase coupling by maps at the level of individual sensors, and not at the level of sensor-pairs. This is important, because phase coupling at the level of sensor-pairs does not reveal the networks of coupled sensors in a straightforward way, at least not without a priori hypotheses about the (sensor, frequency)-pairs that are involved in these networks.

**Fig 1:**
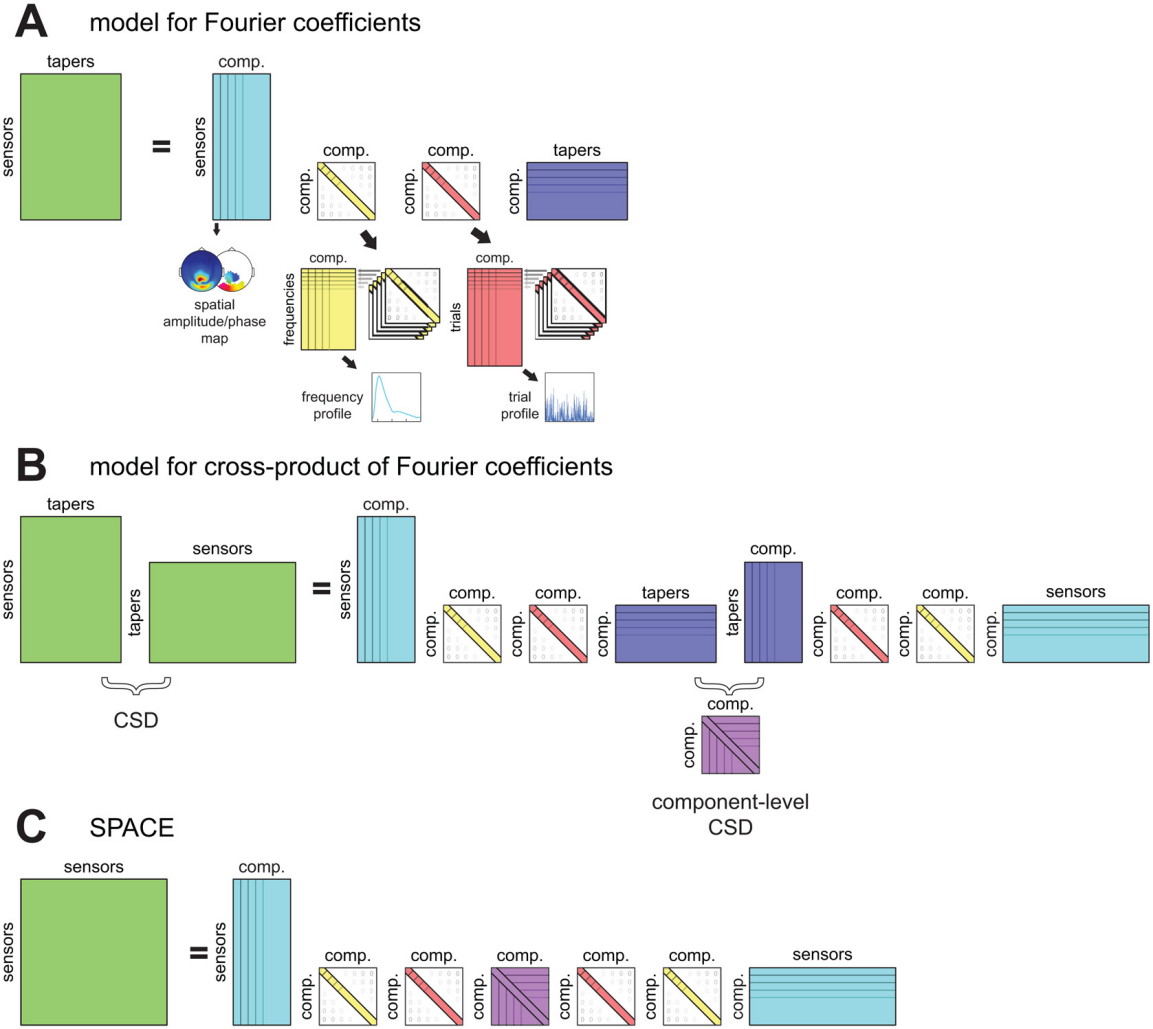
A model for characterizing between-sensor phase coupling by rhythmic components. Our method is based on a model that describes patterns of between-sensor phase coupling of oscillations in electrophysiological recordings. These oscillations are described by Fourier coefficients obtained by a spectral analysis using multitapering, which vary of sensors, tapers, frequencies and trials. Components describe the systematic variability in these Fourier coefficients by a spatial amplitude map, a spatial phase map per frequency, a frequency profile, and a trial profile (see Fig 2 for examples). Here we present the model schematically. We start with a model for the observed Fourier coefficients in A, continue with a model for its cross-product in B, and end with the model we use for extracting components in C. **A.** This model describes the structure in sensor-by-taper matrices of Fourier coefficients (green) per frequency and trial, by the product of four matrices reflecting the four parameter sets. The first (cyan) is complex-valued and contains the spatial amplitude map (see Fig 2B) and frequency-specific spatial phase map (see Fig 2C) for all components. The second matrix (yellow) is real-valued and diagonal, and contains the frequency-specific weighting of components. When combined over frequencies, it forms the frequency profile (see Fig 2A). The third matrix (red) is similar to the second (cyan) but contains the trial-specific loadings, and forms the trial profile (see Fig 2D). The fourth matrix (blue) is complex-valued, and contains the frequency- and trial-specific amplitudes and phases of each taper of each component. **B**. This is a model for the cross products of the matrices of Fourier coefficients. These cross products are the cross-spectral density matrices (CSDs), and describe between-sensor phase coupling averaged over tapers. The model for the cross products is the cross product of the model in A, and contains the same parameters. In the center there are now the component-by-component cross products of the matrices (purple) that contained taper-specific parameters. Importantly, these component-by-component cross products describe the phase coupling between components averaged over tapers, and is the *component-level* CSD. This matrix is explicitly modeled in the final model which we use to extract components. **C**. The final model explicitly models between-component phase coupling by the component-level CSD (purple). Importantly, this is only estimated *per frequency*, and not *per trial and per frequency* (see Materials and Methods for a mathematical description of the model). Components according to this model can be extracted using a method denoted as SPACE.

The decomposition model describes the structure in the frequency- and trial-specific CSDs and is shown in an equation below. As an additional aid, in Fig 1, we also present the model of the CSDs graphically, starting from a model of the observed Fourier coefficients *X*_*jklm*_, and ending with the model shown in the equation below. The parameters of our decomposition model are estimated using an iterative alternating least squares algorithm, which is described in detail in van der Meij, Jacobs [21]. In short, per ALS iteration, each of the four parameters sets described above is sequentially updated using ordinary least squares while the other three parameter sets are held constant. The least squares estimate of the spatial phase maps cannot be determined using ordinary least squares, and is instead determined using new optimization techniques [21]. Once all four sets are updated, an iteration is completed, and the increase in fit is determined. Once subsequent iterations no longer increase the fit beyond a predetermined relative criterion, the algorithm is said to have converged and is stopped. The model is as follows: $X_{kl}\cdot X_{kl}^{*}\;=\;AL_{k}\cdot \text{diag}B_{k}\cdot \text{diag}C_{l}\cdot D_{k}\cdot \text{diag}C_{l}\cdot \text{diag}B_{k}\cdot AL_{k}^{*}+{Ε}_{kl}$. The CSDs ($X_{kl}\cdot X_{kl}^{*}$, green in Fig 1C) are modeled as a product of four matrices, of which three appear twice (*AL*_*k*_, diag *B*_*k*_ and diag *C*_*l*_) and one appears only once (*D*_*k*_). The difference between the model and the observed CSD is the error term *E*_*kl*_. The sensor-by-components matrix *AL*_*k*_ (*J* × *F*, with number of components *F*, cyan in Fig 1C) is complex-valued, and is formed by the spatial amplitude map (specifying the amplitudes of the complex numbers for each sensor *j*) and the frequency-specific spatial phase map (specifying the phases of the complex numbers) of each component. Matrix diag *B*_*k*_ (*F* × *F*, yellow in Fig 1C) is diagonal and real-valued, and contains a weighting of components at frequency *k*. When concatenated over frequencies, the diagonals form the frequency-by-components matrix of frequency profiles *B*. Matrix diag *C*_*l*_ (*F* × *F*, red in Fig 1C) is also diagonal and real-valued, and contains the weighting of components in a trial *l*. When concatenated over trials, the diagonals form the trial-by-components matrix of trial profiles C. Contrary to the matrices that describe components (*AL*_*k*_,diag *B*_*k*_ and diag *C*_*l*_), matrix *D*_*k*_ (*F* × *F*, purple in Fig 1C) describes relations between components. Matrix *D*_*k*_ is complex-valued and conjugate symmetric, and describes the phase coupling between components at frequency *k*. Matrix *D*_*k*_ is denoted as a between-component coherency matrix. Although these matrices are estimated together with the four per-component parameter sets, we do not use them to characterize the components. In fact, they only play a role in a second-level of analysis pertaining to the interactions between rhythmic components (see next section). Importantly, per frequency, only a single spatial phase map is estimated for each component. Therefore, the extracted rhythmic components are only able to reflect oscillatory activity with stationary between-site phase relations over. If the recordings reflect oscillatory activity with dynamic between-site phase relations, then these phase relations can only be captured by extracting multiple components (each capturing a stationary set of phase relations).

**Fig 2:**
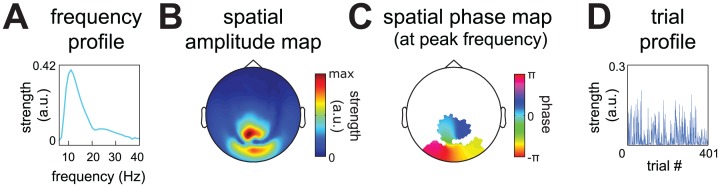
An example rhythmic component reflecting a posterior alpha source. Our decomposition method describes patterns of between-sensor phase coupling over frequencies and over trials by rhythmic components (see also Fig 1). These components are extracted from Fourier coefficients of electrophysiological recordings, which are obtained by a spectral analysis using multitapering. Components describe the systematic structure of Fourier coefficients over sensors, tapers, frequencies and trials and consist of four parameters sets. The frequency profile (**A**) describes the frequencies at which there is between-sensor phase coupling. The spatial amplitude map (**B**) describes which sensors have consistent between-sensor phase relations. The spatial phase maps (**C**) describe, per frequency, the between-sensor phase relations. The trial profile (**D**) describes how strongly a component is present in each trial by a single number. Importantly, the spatial amplitude map and the spatial phase maps describe phase coupling at the level of sensor-pairs, by maps at the level of individual sensors. The component depicted is a posterior alpha component, and is one of 15 components extracted from the representative dataset (see Results).

It is crucial to observe that we are not decomposing a *2-way matrix* of Fourier coefficients, but a *4-way array* with the following dimensions: sensors-by-sensors-by-frequencies-by-trials. This higher dimensionality provides additional structure that can be used for identifying and separating components. The model underlying our method shares this aspect with other N-way decomposition models such as PARAFAC/PARAFAC2 [13, 14, 24] and Shifted CP [20]. Crucially, this higher number of dimensions (N>2) is what allows for extracting components that are unique *without enforcing statistical constraints*. This contrasts with the widely used ICA and PCA, which require non-biological constraints (statistical independence, orthogonality, maximal variance) to ensure uniqueness.

At this point, it is useful to make a technical statement related to our decomposition method as described in van der Meij, Jacobs [21]. Although the underlying model is for the frequency- and trial-specific CSDs, the decomposition method (i.e., the alternating least-squares algorithm) takes as its input the square root of these CSDs. From the perspective of the decomposition method, the square-root CSD is equivalent to the sensor-by-taper matrix of Fourier coefficients, and the latter matrix is the starting-point of the method developed in van der Meij, Jacobs [21]. In this paper, we do not have this equivalence because we have estimated the CSDs in a way that better reflects rhythmic sources (as compared to their usual estimate, see further). Therefore, in the present paper, the square root of our CSD estimate is not equivalent to the sensor-by-taper matrix of Fourier coefficients.

The model underlying our method has a number of trivial indeterminacies, which are resolved by normalizations that do not affect the interpretation of the components. These indeterminacies are similar to the permutation and scaling indeterminacies of PARAFAC/PARAFAC2 [13, 14, 19, 24, 25] and result in ambiguities with respect the absolute amplitude, sign, and absolute phase of the parameters. These ambiguities have been described previously [21], and here we only describe the required normalizations. The absolute value of all real-valued parameter sets is undetermined, and therefore the spatial amplitude maps, the frequency profiles, and the trial profiles, are normalized to have a vector norm of 1. Additionally, the between-component coherency matrices are normalized to have ones on their diagonal. That is, they are constrained to be coherency matrices. The signs of the spatial amplitude maps, frequency profiles, and trial profiles, are undetermined as well, and this indeterminacy is resolved by restricting them to be positive. The absolute phase of the spatial phase maps is also undetermined, and they are normalized per frequency such that the strongest sensor in the spatial amplitude map has a phase of 0. Because of the above normalizations, a component-specific scaling parameter is extracted, which will be denoted as *component strength*, but which does not play a role in the interpretation of the individual components. Because of the different normalizations, the absolute amplitudes and absolute phases are not meaningful. Crucially however, amplitude *ratios* between sensors, frequencies and trials, and phase *differences* between sensors are not affected by these normalizations, and they therefore reveal important characteristics of the sources that are reflected by the components.

The decomposition method is an iterative algorithm that starts from a random initialization of the parameter values. Importantly, the algorithm can converge to a local minimum of the least squares loss function if the random initialization was unfortunate. Such suboptimal decompositions can be avoided by using multiple random initializations. Then, when the algorithm repeatedly finds the same optimal solution from different random initializations, it can be assumed that the global minimum has been found.

The decomposition method is publically available in a GitHub repository termed *nwaydecomp* (www.github.com/roemervandermeij/nwaydecomp). Additionally, it will be made available through the FieldTrip open-source MATLAB toolbox [26], together with a tutorial on its use.

#### An analysis pipeline for extracting rhythmic components

**Fig 3:**
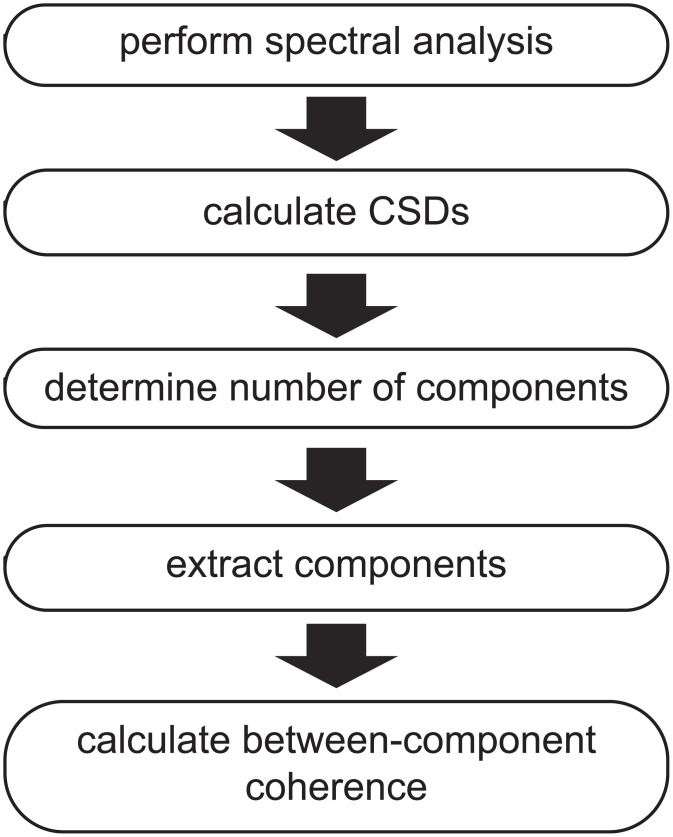
An analysis pipeline for extracting rhythmic components. Extracting components using our method is achieved in several steps, which we present as an analysis pipeline. Electrophysiological recordings are obtained from multiple sensors and multiple trials, and segmented in temporal epochs that reflect trials (or other). A spectral analysis is performed, and subsequently the cross-spectral density matrices (CSDs) are computed, resulting in CSDs per frequency and trial. Once the CSDs are obtained, components can be extracted. The number of components needs to be determined first (e.g. using split-half reliability approach, see Materials and Methods). After extracting the final components, between-component coherence can be estimated. See Results and S1 File.

Extracting components using our method described in the previous section is achieved in several steps, which we present as an analysis pipeline (Fig 3). The inputs for this pipeline are multi-sensor electrophysiological recordings obtained in different trials (also called epochs). Although we demonstrate our analysis approach in the Results by extracting components from MEG recordings, in principle, any type of multi-sensor electrophysiological recordings can be used. In fact, we initially applied our method to ECoG data [21]. Additionally, any type of epoch is suitable, as long as they allow for a meaningful comparison of the epoch-specific component strengths.

We start with a spectral analysis, and compute a CSD matrix for each frequency and trial. We calculate these CSDs in a way that makes them more sensitive to rhythmic sources than regular CSDs (see further). Using the CSDs as input, we extract components in three steps. First, the number of components to be extracted is determined on the basis of an assessment of the components’ reliability. This approach involves splitting the trials in two sets, extracting components from the CSDs of the two sets (using multiple random initializations of the algorithm), and evaluating the components’ reliability by comparing them between the two sets (see further). In the second step, the component-specific parameters are estimated: the spatial amplitude and spatial phase maps, frequency and trial profiles (again using multiple random initializations). Optionally, in a third and last step, we estimate the between-component coherency matrices. This is a necessary step to investigate so-called strong and weak phase-coupled oscillatory networks (PCNs), which are introduced in the Results. Differentiating between these two types of PCNs provides insights into the nature of the communication between interacting sources.

#### Estimating CSDs with greater sensitivity to rhythmic sources

We extract components from frequency- and trial-specific CSDs. Typically, these CSDs are estimated as the cross product of the sensor-by-taper matrices of Fourier coefficients, divided by the number of tapers. Here, we use an alternative estimator, which produces CSDs that are more strongly affected by rhythmic sources. The rationale of this approach, and its benefits, are described in Fransen, van Ede [27]. The alternative estimator capitalizes on the fact that the phase of rhythmic activity is predictable from one cycle to the next. This phase predictability is gradual, and this alternative CSD estimator reflects neuronal activity in proportion to its degree of phase predictability. This also applies to phase coupling *between* neuronal sources: our CSD estimator reflects coupling between neuronal sources in proportion to the degree of predictability of their *phase differences*.

The calculation of the alternative CSD estimator involves a number of steps. In the first step, for a given frequency *k*, the *l*-th trial is cut into *M* non-overlapping segments, which have a length of three cycles of the frequency of interest. Next, each of these *M* segments is tapered with a Hanning window and the Fourier coefficients are calculated for the frequencies of interest (using a Hanning-tapered three-cycle complex exponential). This results in *M* column vectors *X*_*klm*_, each having as many elements as the number of sensors *J*. The regular CSD estimator for frequency *k* and trial *l* involves taking the average of the cross-products $X_{klm}\cdot X_{klm}^{*}$ over the *M* segments (tapers). Now, our alternative CSD estimator is based on the cross products between vectors of Fourier coefficients obtained from adjacent non-overlapping segments:

$$\frac{1}{M-1}\overset{M}{\underset{m\;=\;2}{\sum }}X_{klm}\cdot X_{kl\left(m-1\right)}^{*}$$

Because the cross product is taken between vectors of Fourier coefficients that are based on segments involving a time lag (such that they are non-overlapping), this estimator is denoted as the *lagged CSD* estimator. The crucial advantage of the lagged CSD over the regular estimator based on the cross-products $X_{klm}\cdot X_{klm}^{*}$, is that the former depends on the phase consistency between the adjacent segments, that is, on its phase predictability. In fact, the higher the phase predictability of oscillatory activity, the more this activity is reflected in the lagged CSD. Conversely, the smaller its phase predictability, the less it will be reflected in the lagged CSD.

The above describes the simplest version of the lagged CSD. For our analyses, we calculated a slightly more complicated version, which is based on the same intuition, but makes more efficient use of the data. In its simplest version, the lagged CSD involves non-overlapping segments tapered with Hanning windows, which doesn’t optimally use the segment’s edges. To compensate for this, we cut the trials in segments that have a 75% overlap, and from these segments we selected all segment-pairs whose members were adjacent and non-overlapping. The lagged CSD was then calculated by averaging the cross product over these segment-pairs. Because each of the multiple segments per trial is multiplied with a single taper, this approach is equivalent to a Welch multitapering approach [22]. Although not applied in the present study, one can in principle also multiply each segment with *multiple* tapers (e.g. using Slepian tapers) [23]. In this case, the above cross products are computed only between adjacent non-overlapping segments that were multiplied with the same taper.

Unlike the regular CSD, the lagged CSD is in general not conjugate symmetric and positive semi-definite, although in practice the difference can be small. This is relevant, because our decomposition algorithm requires the CSDs to have these properties. To deal with this, we approximated every lagged CSD by another matrix that does have the required properties. This approximation involves three steps. In the first step, we pre-multiply every lagged CSD by a diagonal matrix that shifts the phases of the rows such that the diagonal elements (which correspond to power in a regular CSD) are real-valued. In the second step, we make the matrix (denoted by *Z*) conjugate symmetric by means of the following transform: *Z* = (*Z* + Z*)/2. Finally, in the third step, we make this matrix positive semi-definite by performing an Eigen decomposition and replacing all negative eigenvalues by zeros. The resulting matrix is conjugate symmetric and positive semi-definite, and will be referred to as the CSD in the remainder. Importantly, compared to the regular CSD, this CSD better reflects sources that are highly rhythmic, as indexed by their phase predictability.

#### Determining the number of components to extract

The number of components in the data cannot be determined analytically and needs to be determined empirically, as is also the case for methods such as PARAFAC and ICA. Rather than attempting to estimate the true number of components, we estimate the number of *reliable* components. For this, we need a reliability index for each of the extracted components. We use a reliability index that is based on an odd-even split-half of the trials. More precisely, after splitting the trials in two halves, we extract components from both halves, and evaluate their between-half similarity. The number of components is increased until the components start to differ between the two halves.

For the analyses presented in this paper we evaluated component reliability by means of split-half similarity coefficients for the spatial amplitude maps, the spatial phase maps and the frequency profiles. These coefficients range between 0 and 1, and we considered component reliability to be acceptable if these coefficients exceeded 0.5 for all three parameter sets. Because multiple components were extracted from both halves, components had to be matched between halves. This was done using the same similarity coefficients.

The split-half similarity coefficients were different for the three parameter sets. For the spatial amplitude maps and the frequency profiles, split-half similarity was calculated as the inner product of, respectively, the normalized spatial amplitude maps and the normalized frequency profiles. For the spatial phase maps, we calculated a split-half similarity coefficient that involves weighting by the spatial amplitude maps and the frequency profiles. This weighting scheme ensures that the similarity coefficient is mainly determined by the most reliable phase estimates, which are obtained from the (sensor, frequency) pairs with the highest amplitudes. This split-half similarity coefficient was calculated as follows:

$$\text{split-half similarity}:\;\frac{\sum_{k\;=\;1}^{K}\left[\left|\frac{A^{1}\cdot exp\left(i\lambda_{k}^{1}\right),\;A^{2}\cdot exp\left(i\lambda_{k}^{2}\right)}{A^{1}\cdot A^{2}}\right|\cdot B_{k}^{1}*B_{k}^{2}\right]}{\sum_{k\;=\;1}^{K}\left[B_{k}^{1}*B_{k}^{2}\right]}$$

The calculation involves two steps. First, we compute the normalized frequency-specific inner product (〈,〉) between the amplitude-weighted spatial phase maps $exp\left(i\lambda_{k}^{s}\right)$ of both split-halves (*s* = 1,2 denotes the two halves of the split-half). In the second step, we take the weighted average of the absolute values of these inner products, where the weights are derived from the frequency profiles *B*^*s*^.

Determining the number of reliable components can be performed using the data at different levels of aggregation. For reasons of computational efficiency, we determined the number of reliable components using data at a higher level of aggregation than the data used for extracting the final components. That is, we determined the number of components on the basis of the trial-averaged CSDs, whereas the final analysis was based on the trial-specific CSDs.

### Application to an MEG Experiment on Cued Tactile Detection

#### Experimental paradigm and analysis of MEG recordings

We analyzed MEG recordings of 11 subjects (4 male; aged 22–49 years) that were obtained and analyzed previously [28]. Each subject participated in two consecutive sessions, resulting in 22 recordings. The study was conducted in accordance with the Declaration of Helsinki, was approved by the local ethics committee (CMO Regio Arnhem-Nijmegen), and all subjects provided written informed consent.

Subjects performed a cued tactile detection task in which the target stimulus was either cued or uncued. The central event in each trial was an auditory stimulus (50ms, white noise) that was paired with a tactile stimulus in half of the trials (0.5ms electric pulse around threshold intensity). The tactile stimulus was delivered to the left or right thumb, and its location was cued by an auditory stimulus (150 ms) on a third of the trials. This auditory cue always preceded the tactile stimulus by 1.5s, and indicated location by pitch (500/1000Hz, counterbalanced over subjects). Subjects indicated on each trial whether a tactile stimulus was present or absent by pressing a button with their index finger after they had received a lateralized auditory response cue. This cue always arrived 1s after the auditory stimulus, and was presented either to the left or the right ear (alternating over trials; 150ms at 1000Hz). To indicate the presence of a tactile stimulus, subjects responded with their ipsilateral index finger, and to indicate its absence they responded with their contralateral index finger. Subjects received auditory feedback indicating accurate detection or not (50ms up-going or down-going frequency sweeps resp.). Cued and uncued trials were randomly interleaved, and subjects completed ~500 trials per recording session. Inter-trial intervals ranged between 2.5 and 12s (mean 3.5s) and were drawn from a truncated negative exponential distribution. For additional details, see van Ede, Koster [28].

For the purpose of the current analyses, we split each trial into three periods. As we had 2 recording sessions from each of the 11 subjects, this resulted in 66 datasets that were analyzed separately. The first period is called *prestimulus*, lasts from t = -1.5s (cue onset) to t = 0s (stimulus), and reflects preparation for the possible arrival of the tactile stimulus The second period is called *stimulus*, lasts from t = 0s (stimulus) to t = 1s (response cue), and reflects the processing of the stimulus (which may or may not be presented). The third period is called *response*, lasts from t = 1s (response cue) to the response button press, and reflects preparation and execution of the response. The duration of this period varied from trial to trial, but was at least 1s long. The average median reaction time over datasets was 1699ms (SD = 255ms) (2 trials were discarded from one dataset because the reaction time was less than 1000ms).

Recordings were obtained from an MEG system with 275 axial gradiometers (CTF MEG; MISL, Coquitlam, British Columbia, Canada), which was housed in a magnetically shielded room. All recordings were low-pass filtered with a 300Hz cutoff and sampled at 1200Hz. Trials containing artifacts were removed using a semi-automatic detection procedure. Power line noise was removed with a discrete Fourier transform filter. Prior to spectral analysis, we removed the mean and the linear trend from each trial. Next, to suppress the 1/f^x^ shape of the power spectrum, the data was prewhitened by taking the first temporal derivative. All preprocessing and spectral analysis (described below) was performed using custom analyses software and the FieldTrip open-source MATLAB toolbox [26].

To extract components, we followed the analysis pipeline described earlier in the Materials and Methods. CSDs were estimated for frequencies between 6 and 40Hz in equally spaced 1Hz bins. We used Welch multitapering, and calculated them with greater sensitivity to rhythmic sources, as described previously. We determined the number of components using the approach described before, in which we randomly initiated the algorithm three times. The final components were extracted from the trial-specific CSDs using five random initializations.

#### Characterizing the spatial and spectral content of components

To characterize the spatial and spectral content of the extracted components, we performed several analyses on the spatial amplitude maps and the frequency profiles. These analyses involved classifying components based on their frequency profile and their spatial amplitude maps.We now describe these analyses in more detail.

We classified components as either alpha, beta, and gamma components on the basis of the peak frequency of their frequency profile (with a range between 6 and 40 Hz). Alpha components had a peak frequency between 8 and 16Hz, beta components had a peak frequency between 16Hz and 30Hz, and gamma components had a peak frequency above 30Hz. Note, these ranges indicate only the range in which the peak could fall and do not reflect the width of the frequency content of the respective frequency profiles. Eight components from eight different datasets of four different subjects had a peak frequency <8Hz or had a frequency profile that was not uni-modal. These components were discarded, and the remaining 783 components were used for further analyses.

To characterize the spatial diversity of components we categorized them on the basis of their spatial amplitude maps. Every component was assigned to one of the following categories: posterior, left and right sensorimotor, anterior, bilateral, and a rest category. Categorization was based on three measures that each reflect a particular aspect of the component’s location on the MEG helmet. These measures make use of a 2-dimensional sensor layout that was constructed from the 3-dimensional sensor positions (identical for all datasets). The first measure is sensitive to the difference between the right and left side of the MEG helmet, and is calculated as follows:

$$\frac{\sum_{j\in \mathbb{R}}\left(A_{j}\right)-\sum_{j\in L}\left(A_{j}\right)}{\sum_{j\in \mathbb{R}}\left(A_{j}\right)+\sum_{j\in L}\left(A_{j}\right)}$$

Here, *A* is the spatial amplitude map and R and L are index sets for the right and the left sensors respectively (132 and 131 sensors resp., remaining sensors were discarded). This measure ranges from -1 (only left sensors contributed to the spatial amplitude map) to 1 (only right sensors contributed). The second measure was constructed in the same manner, but is sensitive to differences between anterior and posterior sensors. For this anterior-posterior measure, we used a sensor in the middle of the helmet to split all sensors in an anterior and a posterior set (125 and 148 sensors resp.). The third measure was also constructed in the same manner, but was sensitive to the difference between sensors close to the anterior-posterior midline (medial sensors) and sensors on the lateral sides of the helmet. Sensors were considered to be medial (146 sensors) when they were located in between two marker sensors, of which one was on the left side and the other was on the right side of the helmet; sensors were considered to be lateral when they were located to the lateral side of these marker sensors (126 sensors). These three measures were used to spatially categorize the components. Boundary values for these measures were determined by visual inspection. Components were categorized as posterior when they had an anterior-posterior value between -1 and -0.3. Left sensorimotor components had a left-right value between -1 and -0.2, a medial-lateral value between -0.25 and 0.25, and an anterior-posterior value between -0.2 and 0.4. Right sensorimotor components were defined similarly, but had a left-right value between 0.2 and 1. Anterior components had a left-right value between -0.2 and 0.2, and medial-lateral and anterior-posterior values between 0 and 1. Lastly, bilateral components had a left-right value between -0.2 and 0.2, a medial-lateral value between -1 and -0.1, and an anterior-posterior value between -0.3 and 0.3.

To show representative examples from each category, we selected the 10 components with the most typical spatial amplitude map, also denoted as representative spatial maps. These components were selected in a stepwise procedure, in which we selected the component that had the highest summed partial correlation with the other spatial amplitude maps (summed over components). The stepwise nature of this procedure follows from the fact that the variance of the already selected spatial amplitude maps was partialled out when calculating the partial correlations. This stepwise selection procedure was continued until 10 components were selected.

#### Statistically testing correlations between contrasts over clustered observations

In the Results, we report on a statistical test involving a correlation over components between two contrasts: cued versus uncued and hit versus miss. These contrasts are calculated at the level of the extracted components. Crucially, multiple components were extracted from the same dataset and therefore cannot be considered independent observations. Instead, these components exhibit a form statistical dependence that is typically denoted as clustered observations (clustering within a dataset). We solved this problem by performing the statistical test at the so-called *second level*, the level of the dataset (also called the *random effects* approach). More specifically, we first calculated the correlations between the two contrasts, separately for each of the datasets (i.e. over the components extracted from this dataset). We then performed a one-sample t-test of the null hypothesis that the average correlation (obtained by averaging over the datasets) was equal to zero.

## Results

In the following, we first briefly describe how a rhythmic component characterizes neuronal activity. Subsequently, we characterize the many rhythmic components that were extracted from MEG recordings during a cued tactile detection task [28]. Using these components, we highlight that our approach reveals more than conventional pairwise power or coherence analyses. We also demonstrate that trial-level quantifications of components are a useful tool to investigate task modulations of neuronal activity. As an example case, we will show that this can provide novel insights into the roles of alpha and beta oscillations in attention. Finally, we will briefly focus on how rhythmic components can be used to investigate so-called phase-coupled oscillatory networks.

### A rhythmic component describes neuronal activity by its spectral content, spatial distribution, and strength across trials

Rhythmic components describe the structure in these Fourier coefficients, where each component consists of four parameter sets, as illustrated in Fig 2. The *frequency profile* (Fig 2A) describes which frequencies are involved in the phase coupling. The *spatial amplitude map* (Fig 2B) describes the degree to which the different sensors reflect the source that is described by the component. The *spatial phase maps* (Fig 2C) describe, per frequency, the between-sensor phase relations that are induced by this source. Finally, the *trial profile* (Fig 2D) contains the component strength in each trial, which can be used to compare conditions.

### Rhythmic components reveal the spatial and spectral distributions of overlapping neuronal activity in extracranial recordings

**Fig 4:**
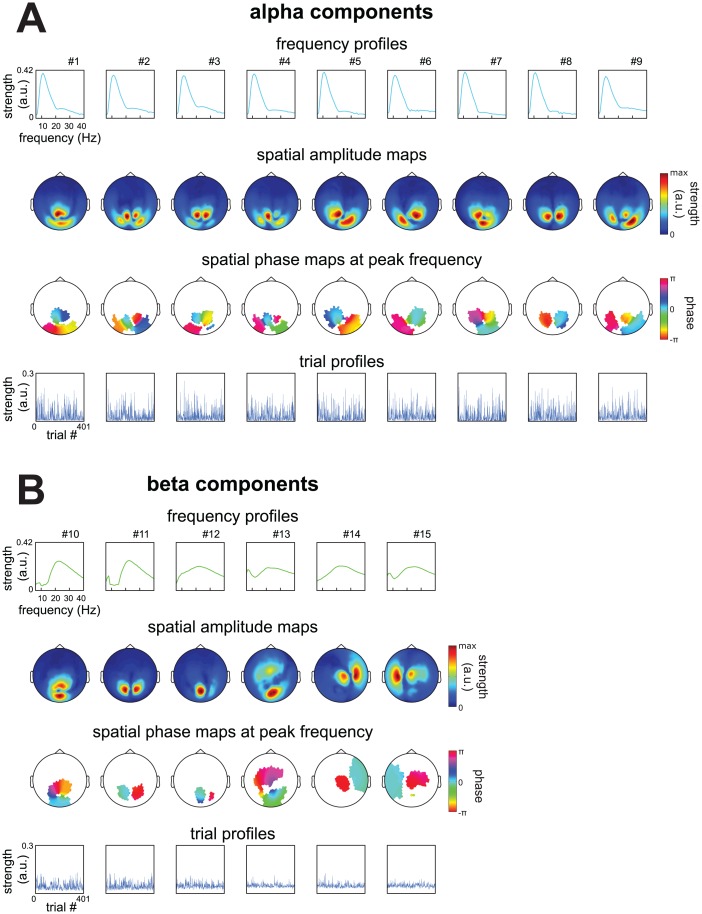
Spatial and spectral structure of sources described by 15 components from a representative subject. We extracted 15 components using the analysis pipeline described in Materials and Methods from MEG recordings of a representative subject performing a cued tactile detection task (see Materials and Methods). This dataset came from the prestimulus period. **A**, frequency profiles, spatial amplitude maps, spatial phase maps at the peak frequency, and trial profiles of components reflecting alpha sources. Phases in the spatial phase maps were masked when amplitudes in the spatial amplitude map fell below 30% of its maximum. **B**, same as A, but for components reflecting beta sources. The components we extracted consisted of (1) many overlapping posterior alpha components, (2) posterior beta components, (3) an occipito-central/frontal component and (4) left and right sensorimotor beta components. Components reflected different types of sources, for a discussion see Results and Sections 1,2 and 3 in S1 File.

We extracted a total of 783 rhythmic components from 66 extracranial datasets. In this section we show components of a representative subject, and contrast them to conventional pair-wise analyses (see the next section for the analysis of all components). From the prestimulus period of the cued tactile detection task of this subject, we extracted 15 components (Fig 4; see Material and Methods for details). These components reflected 9 alpha sources (Fig 4A; peak within 8 to 16 Hz) and 6 beta sources (Fig 4B; peak within 16 to 30 Hz). These involved 9 strongly overlapping occipital alpha components, 3 occipital beta components, an occipito-central component (#13), and 2 sensorimotor beta components. The components differed with respect to the type of sources they reflect. Some of these likely reflect an oscillating point source characterized by a dipolar pattern of between-sensor phase coupling (e.g., #6, 11, 14, 15). This is indicated by their spatial amplitude map having two peaks (local maxima), and by their spatial phase maps at the peak frequency showing phase relations that were mostly 0 and *π*. There were also components with two peaks in their spatial amplitude maps, but with more phase diversity in their spatial phase maps (e.g., #1, 5, 8, 10, 13; for a discussion of phase diversity, see [29, 30, 31]). Additionally, several components had more than two peaks in their spatial amplitude map (e.g., #2, 4, 9). These last two groups of components likely reflect a distributed source or multiple phase-coupled point sources. We denote these source configurations as phase-coupled oscillatory networks (PCNs) and briefly describe them in a later section.

**Fig 5:**
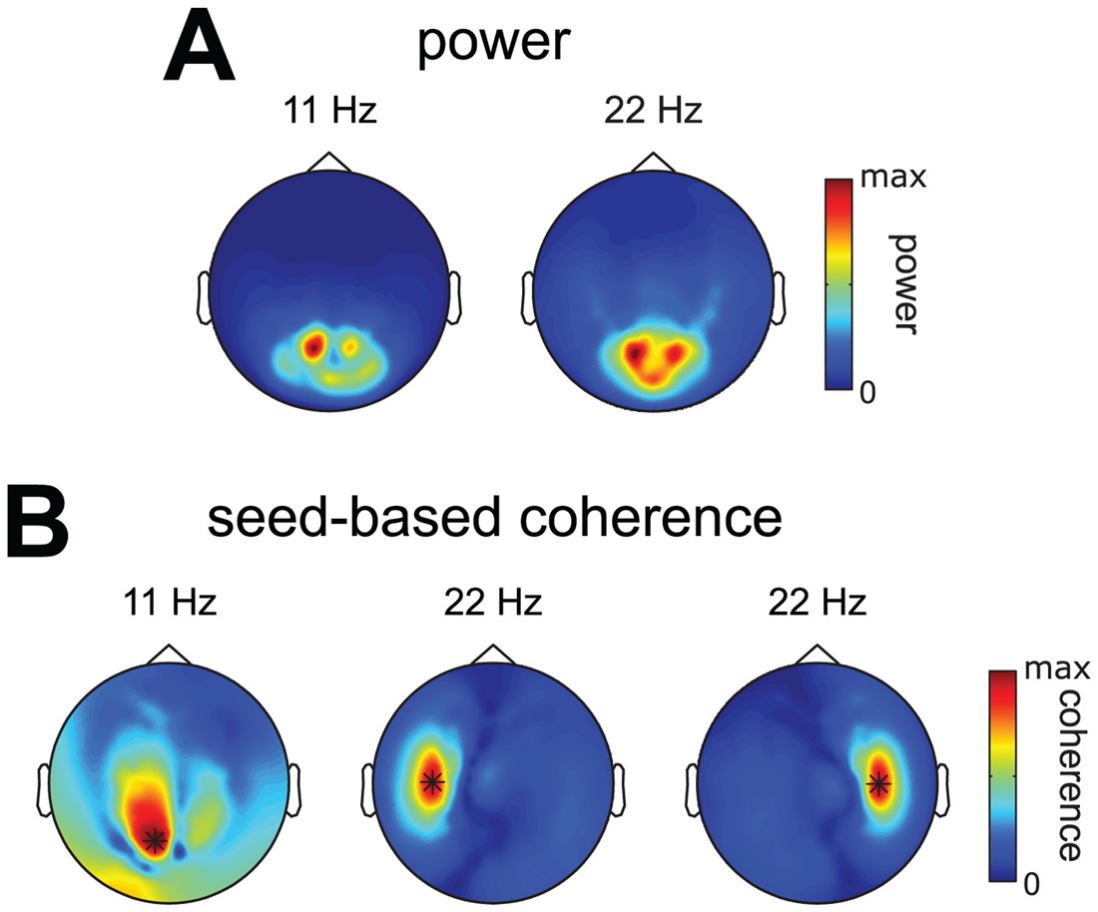
Alpha and beta power and seed-based coherence of the representative subject. To compare the extracted components of our representative subject (Fig 4) to conventional pairwise analyses we computed power and coherence using the same CSDs as were used to extract components. **A**, spatial topography of alpha and beta power. **B**, spatial topography of alpha coherence seed from a posterior sensor, and spatial topography of beta coherence seeded from two sensorimotor sensors. Observe that (1) the analysis of power suggests only occipital activity, (2) occipital seed-based alpha coherence shows distributed coupling over many occipital sensors, and (3) sensorimotor seed-based beta coherence reveals sensorimotor sources with a monopolar pattern. When comparing this to the components extracted using the same CSDs (Fig 4) we observe (1) that occipital alpha power and coherence originated from many overlapping and separable alpha sources, and (2) that there are clear dipolar beta patterns over sensorimotor cortex, strongly suggesting point sources. That these observations cannot be made in power and seed-based coherence illustrates an important point: due to summation of multiple sources sensor-level power and coherence is a very indirect way of studying neuronal activity. Extracting components using our method allows for separating overlapping sources and to analyze them individually.

We investigated whether the alpha and beta sources revealed by the extracted components were also revealed by conventional pairwise analyses involving power and seed-based coherence (Fig 5A and 5B). Power and coherence were computed using the same CSDs that were used for extracting the components. We observe that (1) the analysis of power suggests only occipital activity, (2) occipital seed-based alpha coherence shows distributed coupling over many occipital sensors, and (3) sensorimotor seed-based beta coherence reveals sensorimotor sources with a monopolar pattern. This contrasts with the extracted components, which revealed (1) that occipital alpha power and coherence originated from many overlapping and separable alpha sources, and (2) that there are clear dipolar beta patterns over sensorimotor cortex, strongly suggesting point sources. The lack of dipolar patterns in sensorimotor seed-based beta coherence and the widespread distribution of occipital seed-based alpha coherence, illustrate an important point: as a result of the overlap between the sensor-level signatures of multiple sources, studying sensor-level coherence is a very indirect way of studying distributed patterns of neuronal activity. In contrast, components extracted using our method allows to separate these overlapping sources and to analyze them individually.

In summary, we have shown that the extracted components reflect spatially and spectrally diverse sources that are difficult to identify using conventional pairwise power and coherence analyses. In this example subject, we also showed that the occipital alpha rhythm reflects many separable sources whose spatial maps overlap at the sensor level.

### 783 Rhythmic components reveal spatially and spectrally diverse neuronal activity

We now present the spatial and spectral content of sources reflected by all 783 components that were extracted. In these analyses, we collapse across the three task periods; their differences will be analyzed in the next section. In total, we analyzed 66 datasets (11 subjects * 2 sessions * 3 periods). On average, we extracted 12.0 (SD = 2.0) components per dataset. These components explained on average 50.7% (SD = 6.5%) of the variance in the single-trial cross-spectral density matrices (CSDs) from each dataset.

**Fig 6:**
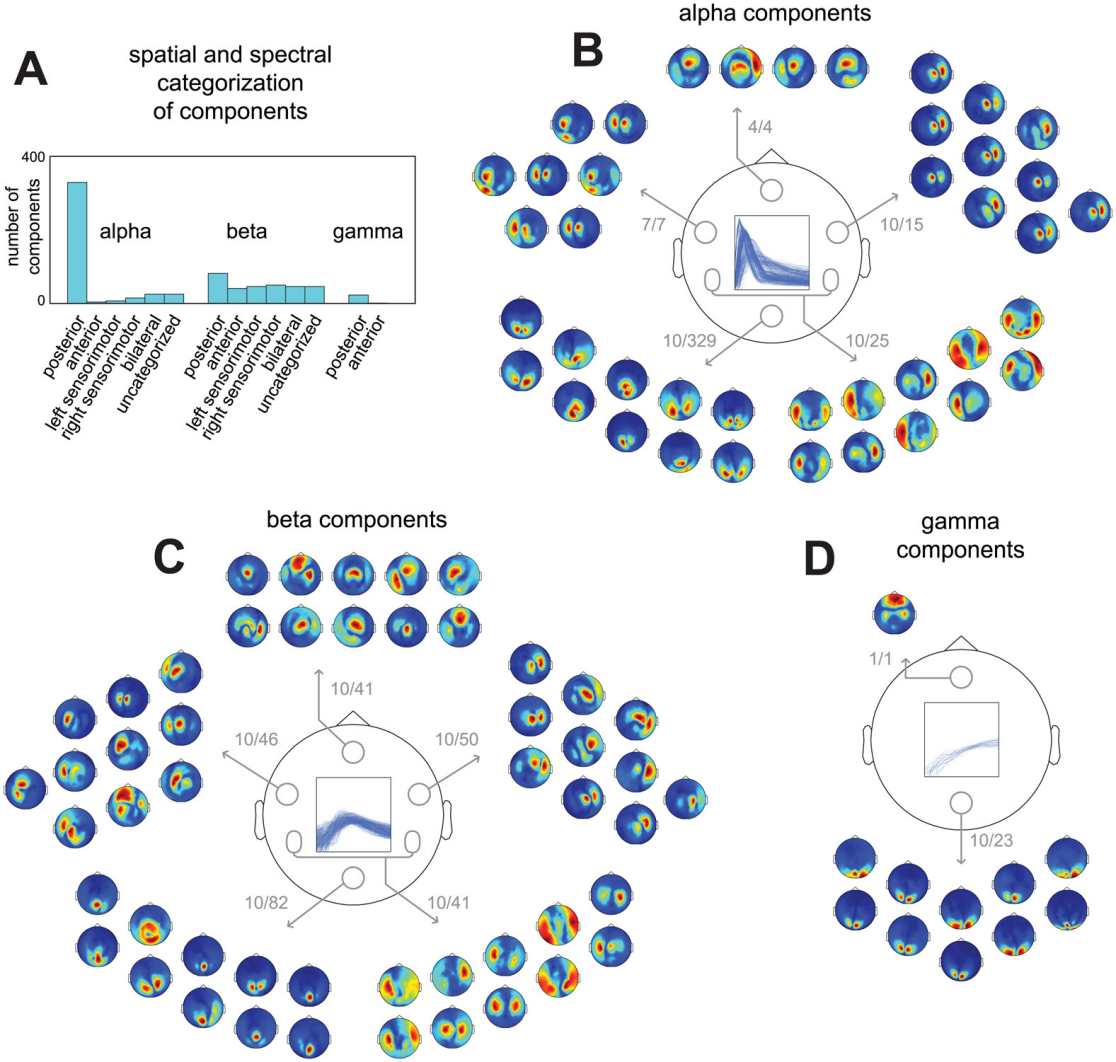
Spatial and spectral diversity of sources described by 783 components extracted from MEG recordings during a cued tactile detection task. 783 components were extracted using the analysis pipeline present in Fig 3 from MEG recordings of 11 subjects in 22 sessions performing a cued tactile detection task, split into 3 trial periods, resulting in 66 datasets. Per dataset we extracted on average 12.0 (SD = 2.0) components, explaining on average 50.7% (SD = 6.5%) of the variance in its CSDs. Based on their frequency profiles we classified 423 components as alpha, 332 as beta, and 28 as gamma components. To visualize their spatial diversity we categorized components based on the distribution of their spatial amplitude maps in 6 categories, describing posterior, anterior, left sensorimotor, right sensorimotor, and bilateral components, and a rest category (see Materials and Methods). **A**, summary of spatial and spectral categorization. **B**, 10 exemplars of alpha components from each spatial category (see Materials and Methods). Numbers indicate the selected and the total number of components in each category. **C**, same as in B but for beta components. **D**, same as in B but for gamma components. From A-D we observe that the extracted components consisted of (1) posterior alpha, beta, and gamma components, (2) sensorimotor alpha and beta components, and (3) some deep (or distributed superficial) alpha but mostly beta components (anterior and bilateral regions).

423 components reflected alpha sources, 332 beta sources, and 28 gamma sources (see Materials and Methods). In order to show the most common spatial distributions across the sensors, we categorized components on the basis of their spatial amplitude maps (Fig 6). We distinguished between six sensor distributions: posterior, anterior, left sensorimotor, right sensorimotor, bilateral, plus a rest category for the remaining components. Components were categorized using their values on three coefficients, each of which indexes their relative location over a particular part of the brain: the left versus the right hemisphere, the anterior versus the posterior part of the brain, and the medial versus the lateral part (see Materials and Methods for details). We first show a summary of this categorization based on sensor distributions (Fig 6A). This reveals a strong difference between alpha, beta and gamma components. For alpha and gamma components, the spatial amplitude maps mostly covered the posterior region. For beta components, on the other hand, the spatial amplitude maps covered all six regions. To show the diversity in the spatial amplitude maps, for five regions (posterior, anterior, left sensorimotor, right sensorimotor, and bilateral), we show the maps of the 10 most representative alpha, beta and gamma components (Fig 6B–6D; see Materials and Methods). From the 10 exemplars in each category, we conclude that the components most likely reflected (1) posterior alpha, beta, and gamma sources, (2) sensorimotor alpha and beta sources, and (3) some deep (or distributed superficial) alpha but mostly beta sources (anterior and bilateral regions).

In summary, we have shown that the components extracted from all datasets revealed numerous alpha and beta sources. As was the case in the representative subject, we also showed that the occipital alpha rhythm is generated by many separable sources whose spatial maps overlap at the sensor level. Next, we used the component’s trial profiles to investigate how the extracted components are modulated by task and behavioral variables.

### Investigating task modulations of neuronal activity by analyzing the components’ trial profiles

As pointed out in the Introduction, it is problematic when sources that overlap at the sensor-level are differentially modulated by some task modulation. Our decomposition method provides a possible solution for this: investigating task modulation of neuronal activity at the component-level. This is possible because the underlying model quantifies in a single number how strongly each component is present in a given trial; combined over all trials, these numbers constitute the *trial profile*. Investigating the relationship between an independent variable and the neuronal activity reflected by a component then reduces to relating this trial profile to the independent variable.

We compare experimental conditions in a cued tactile detection task by comparing condition- and component-specific averages of the trial-level quantifications. We investigate whether the activity reflected by a component is modulated by tactile attention and detection performance. The first contrast compares the average source activity on trials where the location of the stimulus was cued and therefore attended, versus those trials in which it was not cued and thus not attended (cued vs uncued). The second contrast compares the average source activity on trials in which the presence of a stimulus was detected with trials in which it was not detected (hit vs miss). We additionally investigate the relationship between the two experimental contrasts as a function the three task periods, and as a function of the spatial and spectral content of the components.

**Fig 7:**
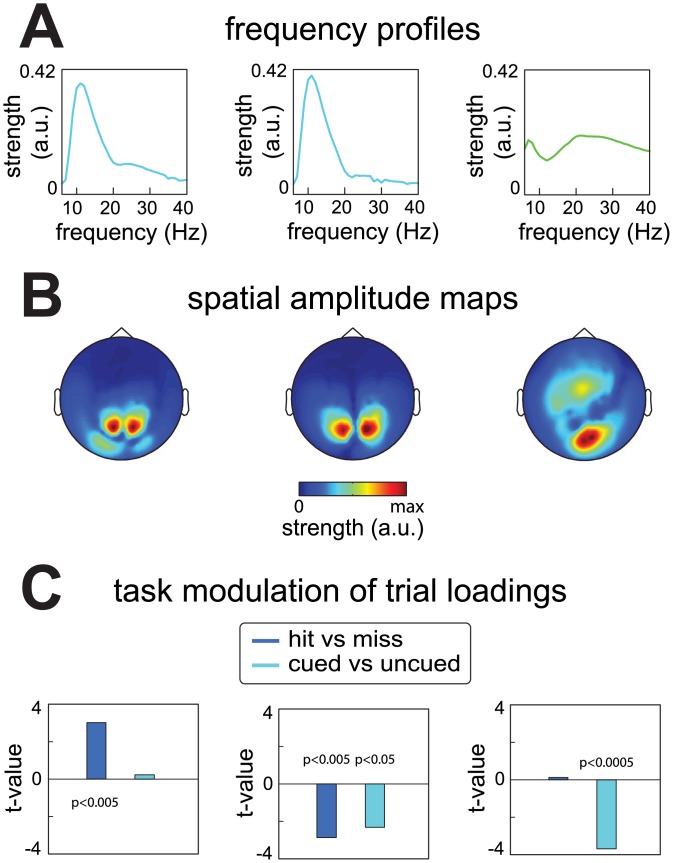
Task modulation of neuronal activity revealed by the trial profile of components extracted from the representative subject. Components reflect neuronal sources, and their strength in each trial is quantified by a single number, combined into the trial profile. These trial-level quantifications can be used to analyze neuronal activity as a function of the task at the level of components. We demonstrate this in three components from the representative subject. The three components were selected out of the 15 because they showed the strongest effects, and reflected two posterior alpha components, and one occipito-central/frontal beta component. **A**, frequency profiles of the selected components. **B**, spatial amplitude maps of the selected components. **C**, task-dependent activity of the components reflected by the contrasts expressed as t-values of independent samples t-tests. The first posterior alpha component was more active on hit trials then it was on miss trials (t(179) = 3.01, p<0.005), but did not differ as a function of attention (t(399) = -0.23, p>0.8). The second posterior alpha component was less active on hit trials than on miss trials (t(179) = -2.87, p<0.005), and was also less active on cued trials than on uncued trials (t(399) = -2.31, p<0.05). The beta component did not differ between hit and miss trials (t(179) = 0.12, p>0.9) but was less active on cued than on uncued trials (t(399) = -3.69, p<5*10^^-4^).

We again start by describing the results for the prestimulus period data of the representative subject (Fig 7). Three components (out of 15; shown in Fig 4) were selected that showed the largest experimental contrasts, as described below. These involved two occipital alpha components and one beta component with a spatial amplitude map over occipital and central regions (Fig 7A and 7B). The effects were quantified by means of independent samples t-statistics. The first occipital alpha component was more active on hit than on miss trials (t(179) = 3.01, p<0.005), but did not differ as a function of attention (t(399) = -0.23, p>0.8). In contrast, the second occipital alpha component was less active on hit than on miss trials (t(179) = -2.87, p<0.005), and was also less active on cued than on uncued trials (t(399) = -2.31, p<0.05). Finally, the beta component did not differ between hit and miss trials (t(179) = 0.12, p>0.9) but was less active on cued than on uncued trials (t(399) = -3.69, p<5*10^^-4^). These examples demonstrate that different components can reveal different task modulations of the sources they reflect. These effects were not all captured by conventional analyses of these data [28].

### Rhythmic components reveal multifaceted task modulation of alpha and beta oscillations in a cued tactile detection task

**Fig 8:**
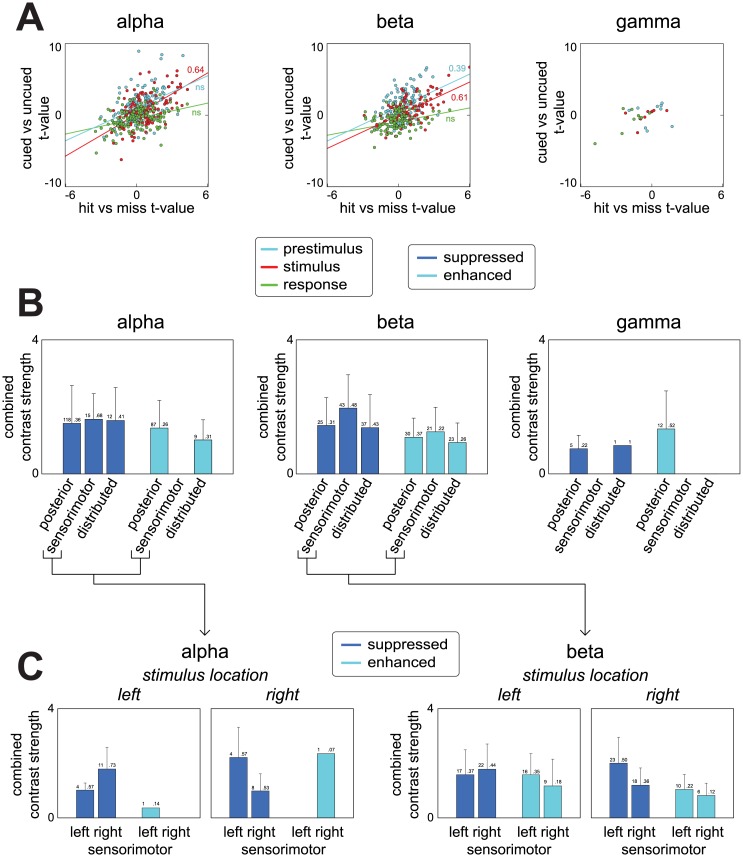
Task modulation of neuronal activity revealed by the trial profiles of all 783 components. We show average source activity contrasted as a function of attention, and subject’s performance on the task, for all 783 components. **A**, attention and performance contrasts expressed by t-values as a function of the three task periods. Lines are obtained via least squares fitting. Activity modulations of alpha and beta components during stimulus processing were positively related: components that were less active on attended trials (cued) were often also less active on trials where the stimulus was detected (hit), and vice versa. Pearson’s correlations were 0.64 (p<5*10^^-6^) and 0.61 (p<5*10^^-4^) for alpha and beta resp. Beta component activity during stimulus preparation showed a similar relation with a Pearson correlation of 0.39 (p<0.005). Correlations for gamma components were not computed due to the low number of components. These analyses revealed that there are sources whose activity is ‘suppressed’ (both less active on cued and hit trials) and ‘enhanced’ (both more active on cued and hit trials). **B**, combined strength of both contrasts as a function of three spatial categories. Bars indicate SD. The spatial categories describe posterior, sensorimotor (‘left/right sensorimotor’), and distributed components. On the top of each bar, we show the number of suppressed/enhanced components present within each category, as well as the proportion w.r.t. the total number of components within each category. **C**, same as in B, but split up for left and right sensorimotor cortex and for trials in which the stimulus arrived on the left thumb or the right thumb. B and C show that there were sources whose alpha rhythms were *suppressed* in both task *relevant* regions (sensorimotor) and task *irrelevant* regions (posterior and others), and that there were sources whose beta rhythms were *suppressed and enhanced* in both task *relevant* regions (sensorimotor) and task *irrelevant* regions (posterior and others). This suggests a complex situation where sources that are located close to each other can be both beneficial and detrimental to the task.

We now turn to all components (Fig 8). To investigate whether task modulation differed between stimulus preparation, stimulus processing, and response preparation/execution, we show the experimental contrasts separately for the three task periods (Fig 8A). Contrasts are again expressed by t-values. For the stimulation period, both alpha and beta components showed a positive relation between the two contrasts in the stimulus period: components whose source activity was weaker on trials in which the stimulus location was cued, also had weaker activity on trials in which the stimulus was detected. Vice versa, components whose source activity was stronger on cued trials also had stronger activity on hit trials. Correlations between the two contrasts in the stimulus period were 0.64 (p<5*10^^-6^) and 0.61 (p<5*10^^-4^) for alpha and beta components respectively. During the preparation period, beta component activity revealed a similar relation between the two contrasts, with the contrasts having a correlation of 0.39 (p<0.005). We did not compute correlations for the gamma components due to the low number of components. As a statistical note, we tested all correlations using a random effects approach (using neuroimaging terminology, *at the second level*), with the components being the first level and the subjects’ datasets the second (see Materials and Methods).

In the above, we show that there are alpha and beta components, whose activity is up- or down-regulated when attention is directed to the location of a possible stimulus, and whose activity is modulated *in the same direction* when a stimulus is correctly detected. On face value, these phenomena have been reported before. First considering down-regulation, knowledge of the location of the stimulus aids in its detection [32], which in task-relevant regions is associated with the suppression of alpha and (in case of somatosensory attention also) beta rhythms. This has been demonstrated both in the visual modality (e.g. [33, 34, 35]), and the somatosensory modality (e.g. [28], involving an analysis of the same data as the current paper, [36, 37]). Next, considering up-regulation, we additionally find alpha and beta components that show the reverse. That is, the activity of these components is higher during attention and when stimuli are detected accurately. This has also been reported before, with alpha rhythms being enhanced and thought to suppress task *irrelevant* regions [38, 39]. Whether our components show suppressions only in task relevant regions (sensorimotor) and enhancements only in task irrelevant regions (posterior and others) is revealed by their location, which we investigate next.

To investigate the distribution across the sensors of the components whose activity was both up-regulated or both down-regulated with attention and performance, we expressed the combined strength of both contrasts as a function of the spatial category of their associated spatial amplitude maps (Fig 8B). These spatial categories were combinations of those described previously and they involve components reflecting posterior, sensorimotor (left and right), and deep/distributed sources (anterior and bilateral). We show the combined contrast strength for two selections of components: those whose sources were down-regulated both on trials where the stimulus could be attended and on trials where it was detected, and those whose sources were up-regulated on both types of trials (as reflected in, both negative and both positive t-values for the two contrasts). We denote these two types of components as, respectively, suppressed and enhanced. Combined contrast strength of a component was obtained by computing the average of the t-values for both contrasts. Of the 423 alpha components, 170 were suppressed and 101 were enhanced. For beta components, this was 133 and 87 out of 332. For gamma components this was 7 and 12 out of 28. Suppressed and enhanced components were present in all three spatial categories.

To interpret the results in Fig 8B it is important to distinguish between components residing in *task relevant* regions and those residing in *task irrelevant* regions. For our somatosensory task, all but the sensorimotor components can be denoted as in task irrelevant regions. The sensorimotor components can both reside in task relevant and irrelevant regions depending on which thumb the stimulus arrived. In Fig 8C we show combined contrast strength separately for trials in which the stimulus was expected on the left thumb and for trials in which it was expected on the right thumb, and do this for left and right sensorimotor components. For the alpha components, Fig 8C shows that, both in task *relevant* (sensorimotor cortices) and task *irrelevant* regions (visual/sensorimotor cortices and others), there are sources whose alpha rhythms are suppressed. Components with enhanced alpha rhythms, however, were only observed over task irrelevant regions. For the beta components, the pattern of results is more symmetrical: both for task relevant and task irrelevant regions, there are both sources whose beta rhythms are suppressed and sources whose beta rhythms are enhanced. Together, these findings suggest a complex situation where sources that are located close to each other can be both beneficial and detrimental to the task. Given that these sources often have overlapping sensor-level representations in extracranial recordings, this complexity would be difficult to appreciate when performing analyses at the sensor-level.

In summary, using a cued tactile detection task as a case study, we have illustrated how rhythmic components can be used to investigate task dependent neuronal activity. We have shown that were many alpha and beta sources whose activity systematically varied with tactile attention and performance. Importantly, these sources reflected either a suppression or enhancement of rhythms in regions both relevant and irrelevant to the task. The signals from these sources often overlapped in our extracranial recordings due to field spread, but our method was able to separate them.

### Rhythmic components allow for investigating phase-coupled oscillatory networks (PCNs) and can distinguish between weak and strong PCNs

A rhythmic component describes the spatial, spectral and temporal structure of phase coupling. Multiple neuronal populations can form phase-coupled oscillatory networks (PCNs). Using our method, it is possible to investigate PCNs. In fact, our initial presentation of the method focused solely on such networks [21]. Using our method, and the between-component coherency matrices described in the Materials and Methods as well as in the Supplementary Methods in S1 File, we can distinguish between two types: strong and weak PCNs. A strong PCN is a spatially distributed neuronal population whose subpopulations (not necessarily continuous in space) are strongly phase-coupled (in the extreme case, with a coherence of 1). A strong PCN is captured by a single component. A weak PCN is a collection of neuronal populations that are only weakly phase-coupled (e.g., only during some parts of an extended period), and is captured by multiple phase-coupled components, each of which reflects one neuronal population. The analyses of these types of PCNs is covered in detail in our Supplementary Methods in S1 File, and we only highlight the key results here. Briefly, strong PCNs were common among the components we extracted from our MEG recordings (Figure A in S1 File), and consisted of both alpha and beta components of both the suppressed and the enhanced types. Weak PCNs however, were exclusively formed between alpha components (Figures B and C in S1 File).

## Discussion

We have presented an analysis approach of oscillatory neuronal activity involving rhythmic components that are extracted according to a new decomposition method. This method characterizes components in terms of their spatial maps (involving both amplitude and phase information), their frequency profiles, and their trial profiles. The novelty of this approach lies in that this method describes the sensor-level representations of sources in a neurobiologically plausible way: a spatially distributed oscillation with between-sensor phase relations that can vary as a function of frequency. In a case study, analyzing MEG recordings during a cued tactile detection task, we demonstrated the following three key benefits of this approach. First, it can separate multiple overlapping sources on the basis of their structure as a function of space (sensors), frequency and time (trials), thereby revealing sources that are very difficult to identify in conventional pairwise analyses. Secondly, it allows for a straightforward analysis of task modulations at the component level, by making use of the trial profiles. Lastly, it can reveal phase-coupled oscillatory networks (PCNs).

### Separately characterizing the activity of overlapping sources

Our method is capable of separating sources using the structure over sensors, frequencies, and trials. Spatially overlapping components are separated on the basis of their different spectral content and trial structure. Similarly, spectrally and temporally overlapping components are separated on the basis of their different spatial maps and, respectively, their different trial structure and spectral content. Separating sources is important because the activity of different sources can be differentially modulated by experimental and behavioral variables. Without separating sources, the activity of overlapping sources is summed, and only their average modulation can be investigated. Moreover, sources differ with respect to the degree with which they are visible at the sensor-level, and this is not only a function of the relevance of a source in an experimental context, but also of unrelated variables such as source depth and the spatial extent of the involved neuronal populations. As a result, the activity of weak sources is hardly visible when these overlap with stronger sources. Using our method, we were able to separate many overlapping sources, such as those that contributed to posterior alpha activity, and we were able to extract sources that were hardly visible at the sensor-level, such as the sensorimotor beta sources (Figs 4 and 5).

Note that *source separation* methods (SPACE, ICA, PARAFAC) have a different goal then *source reconstruction* methods (e.g. beamforming, minimum norm estimation; for a review see [40]). While the latter are capable of resolving the location of a source, they are unable to separate sources with overlapping spatial distributions.

### Analyzing modulations of neuronal activity at the component-level: novel insights on the role of alpha and beta oscillations in attention, as studied in the somatosensory modality

Our method allows for a new way of investigating the relation between neuronal activity, and experimental and behavioral variables. This is normally done by investigating activity at the sensor- and/or source-level, whereas we propose to investigate neuronal activity at the component-level. This is possible because a component’s trial loading is a convenient measure of the trial-specific activity reflected by a component. Using a given component’s trial profile, conditions can be compared at the component-level, namely by comparing its loadings for the different conditions. By analyzing neuronal activity at the component-level, we found that there were many alpha and beta sources whose activity was suppressed or enhanced by attention and in relation to behavioral performance. This occurred both in regions relevant (sensorimotor cortex) and irrelevant (e.g. visual cortex) to our somatosensory attention task. However, studies investigating activity at the sensor-level, which describe the *average task modulation of multiple overlapping sources*, only give a partial view on these effects. Studies investigating alpha activity in attention and memory task mostly report alpha being enhanced in regions irrelevant to the tasks [38, 39, 41–43], and alpha being suppressed in regions relevant to such tasks [28, 33–37, 44]. Likewise, studies investigating beta activity in somatosensory and motor tasks mostly report beta being suppressed in regions relevant to these tasks (e.g., [28, 36, 37, 45–49]). The above findings have led to the view that alpha and beta oscillations in visual and somatomotor regions reflect an inhibition of irrelevant neuronal activity. However, we additionally revealed many alpha and beta sources whose activity was *suppressed* in *irrelevant* regions, and those whose activity was *enhanced* in *relevant* regions. Such alpha and beta sources therefore likely do not reflect inhibition of irrelevant information, suggesting that the functional role of alpha and beta activity in such tasks is more complex than is commonly thought. This highlights the usefulness of a method like ours, which can separately characterize the activity of these different sources.

### Relation between our approach and seed-based approaches

Our method is one of many that have been proposed to identify and characterize networks in electrophysiological signals. We now compare our method to existing ones, which we can group in two categories: (1) seed-based approaches, which we discuss here, and (2) decomposition methods, which we discuss in the next section. For the seed-based approaches, we must further distinguish between methods for identifying networks in resting-state electrophysiological signals [50–52] and methods for identifying networks that are differentially active in different experimental conditions [53]. We start with the former. The seed-based approach starts from a pair-wise measure that indexes the coupling between two sensors. Commonly used measures are coherence [54], phase-locking value [55], and pair-wise phase consistency [56]. It is problematic to investigate phase coupling between *all* sensor-pairs because the result cannot be visualized in a way that is easy to interpret. For this reason, one often selects a seed sensor and visualizes the coupling with all other sensors. This approach requires a priori hypotheses about which (sensor, frequency)-pairs are likely to reveal the networks. In contrast, our approach describes coupling at the level of sensor-pairs by spatial maps at the level of individual sensors, which are more straightforward to interpret.

The seed-based approach is further hampered by the fact that extracranial signals suffer from field spread (volume conduction of electrical fields and common pickup of magnetic fields), which results in spatial overlap between signals from separate sources, as we demonstrate in Figs 4 and 5. In an attempt to deal with this field spread, some authors apply the seed-based approach to signals at the source level [50–53]. That is, they first use source reconstruction methods, such as the beamformer, to calculate source-level signals, and then characterize coupling between a seed-region and all other regions of interest using pair-wise measures. However, source reconstruction is not sufficient to remove all effects of field spread. Exactly for this reason, Hipp, Hawellek [52] use a pair-wise measure that is only sensitive to phase coupling that cannot by fully explained by field spread. This measure is based on the idea that average phase relations different from zero cannot be due to field spread [15]. In contrast, our method can deal with the effects of field spread (1) by separating sources with overlapping sensor-level spatial maps (see e.g. Figs 4 and 5), and (2) by providing spatial phase maps that allow us to distinguish between phase coupling caused by field spread (0 or π) from phase coupling caused by interacting populations (other than 0 or π; see Supplementary Methods in S1 File).

We now consider a seed-based method for identifying networks that are differentially active in experimental conditions. This method was proposed by Hipp, Engel [53]. This method is a statistical method, because it evaluates the statistical significance of differences between conditions. The particular method used by Hipp, Engel [53], cluster-based permutation tests [57, 58], produced contiguous clusters in space, frequency, and time. These clusters were used to identify the networks that were modulated across experimental conditions, as well as the time window in which this modulation occurred. Importantly, because these clusters were defined by a statistical criterion, their size depends on the sensitivity of the statistical test, which in turn depends on the number of observations and its signal-to-noise ratio. Thus, cluster size will increase with, for example, the number of subjects or trials. This is an important constraint on the interpretation of the clusters. In contrast, the spatial extent of our components is determined by how strongly each sensor picks up activity from a source, regardless of the modulation of source strength across experimental conditions.

### Relation between our approach and other decomposition methods

We now consider alternative decomposition methods. It is useful to distinguish between methods that can only decompose arrays of Fourier amplitudes (which are real-valued), and those that can decompose arrays of complex-valued Fourier coefficients. Among the former methods are ICA and PCA [9, 10], and real-valued PARAFAC [11–14]. These methods are useful for identifying networks of neuronal populations with correlated amplitude envelopes. However, it is unclear how the correlation between amplitude envelopes is relevant for the effective communication between neuronal populations. For phase coupling on the other hand, there are plausible mechanisms arguing for their relevance for this effective communication [4, 59, 60]. This is the main motivation for using decomposition methods that characterize networks by their patterns of between-sensor phase coupling in arrays of complex-valued Fourier coefficients.

A few decomposition methods are capable of decomposing complex-valued arrays. These are complex-valued PARAFAC [19], Shifted CP [20], and complex-valued ICA [17, 18]. These methods all characterize phase-coupled oscillatory networks, but suffer from a number of shortcomings that do not apply to our method. These methods (1) require constraints that are not neurobiologically motivated (complex ICA), (2) cannot deal with trial- and frequency-specific phase offsets (complex ICA and PARAFAC), (3) cannot make use systematic variability over frequencies to identify networks (complex ICA), and/or, (4) can only describe between-sensor phase relations that are constant over frequencies (Shifted CP and complex PARAFAC). The last of these shortcomings is of particular interest in relation to our method. It limits the implicated methods to describing phase relations of point sources, because other source configurations need not be limited to phase relations that are constant over frequencies. For example, this is the case for a source whose subpopulations communicate with a time delay, which results in phase relations that increase linearly with frequency (e.g. a traveling wave). A model-based method, similar to the one used in this paper, but targeted towards identifying sources whose subpopulations communicate with time delays, has been described in the same original presentation [21].

## Conclusions

We have presented and applied a novel approach for analyzing oscillatory neuronal activity using a decomposition method that can separate overlapping sources by their patterns of between-sensor phase coupling, their spectral content, and their variable presence over trials. Unlike existing decomposition methods, it uses a plausible model of a neurobiological rhythm: a spatially distributed oscillation with energy in a range of frequencies and involving between-sensor phase relations that can vary over frequencies. Our approach also allows for analyzing task modulations of neuronal activity at the level of these separated sources, which, as we have shown for the exemplary case of a cued tactile detection task, is a powerful alternative to conventional pair-wise analyses. These capabilities make our method a useful tool for investigating the role of oscillatory activity in cognition and behavior, and help untangle the many neuronal interactions that are present in electrophysiological recordings.

## Supporting Information

S1 File Van_der_Meij_et_al_extracting_extracranial_rhythmic_components_SUPPLEMENTARY.pdf (PDF)
