# Supplementary material for: Rhythmic Components in Extracranial Brain Signals Reveal Multifaceted Task Modulation of Overlapping Neuronal Activity
Source: PLoS One. 2016 Jun 23;11(6):e0154881. doi: 10.1371/journal.pone.0154881 (PMC4918966; doi:10.1371/journal.pone.0154881)
Supplement: S1 File — (PDF) [file pone.0154881.s001.pdf]

Supplementary Results

1. Rhythmic components describe both strongly and weakly phase-coupled oscillatory networks (PCNs)
A rhythmic component describes the spatial, spectral and temporal structure of phase coupling that is induced by a neuronal population. Neuronal populations can form phase-coupled oscillatory networks (PCNs) and, using our method, we can distinguish between two types, strong and weak PCNs. A strong PCN is a spatially distributed neuronal population whose subpopulations (not necessarily connected in space) are strongly phase-coupled (in the prototypical case, with a coherence of 1). As we will explain below, a strong PCN is captured by a single component. A weak PCN is a collection of neuronal populations that are only weakly phase-coupled (e.g., only during some parts of an extended period). As we will explain below, a weak PCN is captured by multiple phase-coupled components, each of which reflects one neuronal population. These populations can be point sources or they can also be spatially distributed. In the latter case, this neuronal population is itself a strong PCN. Thus, a weak PCN can be a phase-coupled network of strong PCNs, but may also involve components that reflect point sources.

Strictly speaking, the distinction between distributed and point sources is not biologically realistic. However, it is useful because it allows for making a distinction between components that are strong PCNs (distributed sources) and those that are not (point sources). In practice, we will consider a component to be a point source if its spatial amplitude and spatial phase map closely approximate a current dipole; otherwise, it will be considered a distributed source. Note that a distributed source can be spatially discontinuous, and when the disconnected subpopulations are small, the source's spatial amplitude and spatial phase map can look like a superposition of point sources (e.g., component #2 in Fig 4).

When extracting components, we have to deal with the fact that a single component can be split in any number of components with between-component coherences equal to 1. In terms of the matrices in Figure 1, this splitting of components corresponds to a single spatial map (a column in the leftmost cyan matrix) being replaced by multiple spatial maps, which can be combined into the original one. Importantly, after this split, the corresponding columns of the between-component coherency matrices are equal to each other (and, as a result, the whole matrix is rank-deficient). We prevent such a split-up of components by putting a constraint on the between-component coherencies. Specifically, we constrain these matrices to be identity matrices (zero between-component coherence). Under this constraint, a PCN that exhibits perfect phase coupling between subpopulations will be captured by a single component. In fact, if this PCN would be split over multiple components then some off-diagonal elements of the between-component coherency matrices will be equal to 1, strongly conflicting with the zero coherence constraint. This constraint also has the consequence that groups of sensors that correspond to weakly phase-coupled neuronal populations, will be split into separate components. After estimating the component-specific parameters under the identity matrix constraint, these weak PCNs are identified by estimating the between-component coherencies while keeping the component-specific parameters fixed.

The zero coherence (identity matrix) constraint unavoidably leads to some degradation of the component-specific parameters of phase-coupled components. As such, our strategy is only valid under two assumptions: (1) the resulting component-specific parameter degradation is minimal, and (2) the

between-component coherencies can be estimated reasonably from these parameters. We evaluated the robustness of our strategy by simulating overlapping phase-coupled MEG components, and varying the strength of their coherence. The simulations and their results are described in detail in Supplementary Methods section 1. In short, this simulation study showed that (1) the component-specific parameters are only minimally distorted by the constraint, (2) between-component coherence can be recovered well, but (3) the between-component phase relation cannot.

In the following two sections we first describe strong PCNs that are revealed by single components extracted from our MEG recordings. Next, we will describe weak PCNs revealed by their between-component coherencies.

2. Strong PCNs are widespread in MEG recordings

To identify single-component strong PCNs they need to be distinguished from components that reflect single oscillating point sources. This is achieved by investigating their spatial amplitude and spatial phase maps. A point source has a dipolar pattern at the sensor level: a spatial amplitude map with two peaks, and a between-peak phase relation of π. When a component has more than two peaks and/or it has different between-peak phase relations, it cannot reflect a point source. Instead, such a component reflects a distributed source, or multiple phase-coupled point sources, and thus it reflects a PCN.

We show identified strong PCNs in Figure A in S1 File. These PCNs were identified on the basis of the between-peak phase relation, which were obtained from the component's spatial phase map (at the peak frequency of the frequency profile). Spatial peaks were detected by comparing the loadings of the spatial amplitude map between neighboring sensors (see Supplementary Methods section 3; peaks of representative subject components are shown in Figure B in S1 File). Alpha, beta, and gamma all showed (1) components with two spatial peaks and between-peak phase relations of π, and (2) components with more than two peaks and/or between-peak phase relations different from π (Figure A in S1 File, panel A). Components reflecting beta sources most often had more than two peaks (62%), followed by alpha sources (47%). To investigate the spatial distribution of strong PCNs, we show the peak-to-peak within-component connections of each component reflecting a strong PCN (Figure A in S1 File, panel B). Strong PCNs were identified on the basis of a strategy that excludes point sources: a component was considered a strong PCN if at least one of the between-peak phase differences fell between $-3/4\,\pi$ and $3/4\,\pi$ on the right-hand side of the circle (between 135° and 225°). Alpha, beta and gamma sources all reflected widespread strong PCNs. Beta components reflected strong PCNs more often (60%) than alpha components (50%), and gamma components (18%). Additionally, strong PCNs in the beta band appeared more widely distributed than strong PCNs in the alpha band. These numbers also indicate how many components reflected point sources: in the alpha, beta and gamma band, these percentages were, respectively, 40%, 50% and 82%. To investigate whether the emergence of strong PCNs was task-dependent, we also show strong PCNs separately for the suppressed and the enhanced component types (Figure A in S1 File, panel C). There were strong PCNs both among the suppressed and enhanced component types, and this was the case for both alpha (46% and 54% resp.) and beta (65% and 49% resp.) components. Because of the low number of components, we did not investigate gamma PCNs. In sum, the above results show that strong PCNs (1) are common, (2) widely distributed, and (3), were common both among suppressed and enhanced component types.

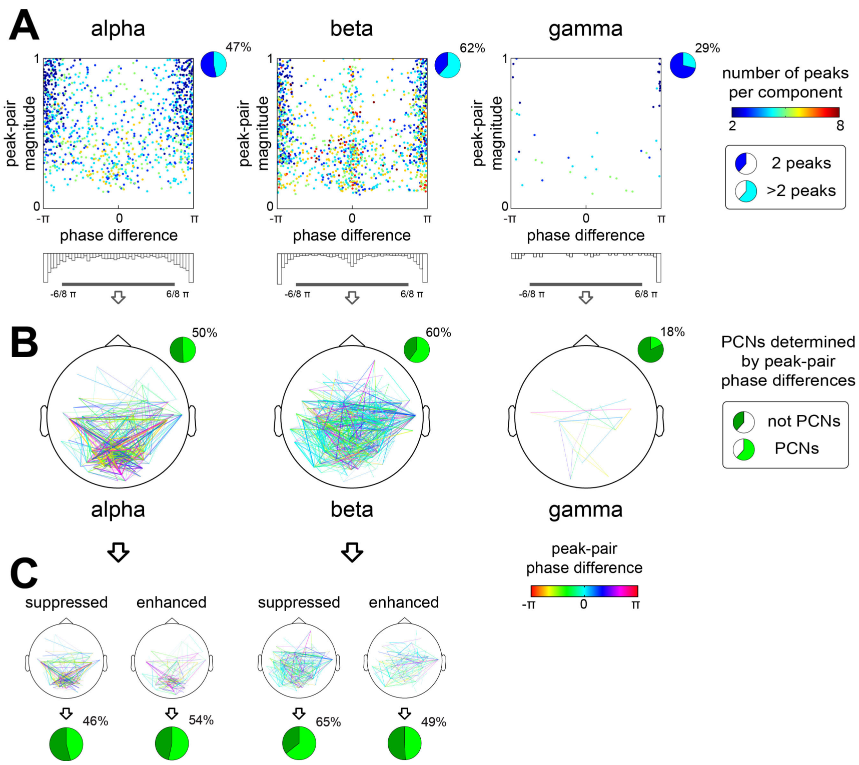

Figure A. **Strongly phase-coupled oscillatory networks (PCNs) are widespread in MEG recordings**. Single components that describe distributed sources reflect strong PCNs, and to identify them they need to be distinguished from oscillating point sources. This is achieved by investigating their spatial amplitude and spatial phase maps. A point source has a spatial amplitude map with two peaks (local maxima), and a between-peak phase relation of π. When a component has more than two peaks and/or it has different between-peak phase relations, it cannot reflect a point source. Such a component describes a distributed source (which can include multiple phase-coupled point sources) and thus reflects a strong PCN. Here we present single-component PCNs identified in components from all datasets. **A**, within-component peak-pair phase relations of the spatial phase map at the peak frequency of the frequency profile. Each dot represents a peak-pair within a component, and is color coded w.r.t. to the total number of peaks in the spatial amplitude map of the component. Peak-pair magnitude is determined by the product of the amplitude of the peaks, with the spatial amplitude maps normalized to have a maximum value of 1. Peaks were detected by comparing the loadings of the spatial amplitude map between neighboring sensors (see Supplementary Methods section 3; example peaks are shown in Figure B in S1 File). Alpha, beta, and gamma all showed (1) components with two spatial peaks and between-peak phase relations of π, and (2) components with more than two peaks and/or between-peak phase relations different from π. **B**, peak-to-peak within-component connections of each component likely reflecting a strong PCN. Line color reflects peak-pair phase difference and line thickness reflects peak-pair magnitude. Components were considered as a PCN when they had at least one peak-to-peak phase difference between $-3/4\,\pi$ and $3/4\,\pi$ on the right hand side of the circle. To avoid showing connections between poles of a dipole we only show connections inside this phase interval. Alpha, beta and gamma sources all reflected single-component strong PCNs and were widely spatially distributed. Beta sources described strong PCNs most often (60%), followed by alpha sources, (50%) and gamma sources (18%). Additionally, beta PCNs appeared more widely distributed than alpha strong PCNs. Non-PCN components reflected point sources, being 40%, 50% and 82%. **C**, peak-to-peak within-component connections as in B but for 'suppressed' and 'enhanced' components separately. Suppressed and enhanced components had either both negative t-values or both positive t-values on the experimental contrasts used in Figure 7 and 8. There were single-component strong PCNs both among the suppressed and enhanced component types, and this was the case for both alpha (resp., 46% and 54%) and beta (resp. 65% and 49%) components. We did not investigate this for gamma components due to their low number. The above results show that (1) single-component strong PCNs are common, (2) are widely distributed, and (3), were common among 'suppressed' and 'enhanced' components.

3. Weak PCNs are revealed by between-component coherence

In Figure B in S1 File, we present weak PCNs formed by components of our representative subject. We show the between-component coherence and spatial distribution for the alpha band weak PCNs in panels A and B. The spatial distribution is shown as connecting lines between the peaks of distinct components (which is different from the visualizations in Figure A in S1 File panel B and C, but the peaks were detected in the same way; see Supplementary Methods section 3). The colors of the connecting lines reflect the strength of their between-component coherence (obtained by averaging between-component coherence over frequencies, weighted by the frequency profiles). Many of the alpha components were coherent and thus reflected weak PCNs. Interestingly, there was diversity in the between-component coherence: coherence between some components was weak, even though their coherence with other components was strong. Surprisingly, no weak PCNs were found in the beta band (Figure B in S1 File, panel C and D).

The weak PCNs that were found in all datasets are shown in Figure C in S1 File. Between-component coherence and its spatial distribution are visualized in the same way as for the representative subject. For alpha components, weak PCNs were common, and were mostly observed over posterior areas. In contrast, beta and gamma components did not show substantial between component coherence, and therefore did not form weak PCNs.

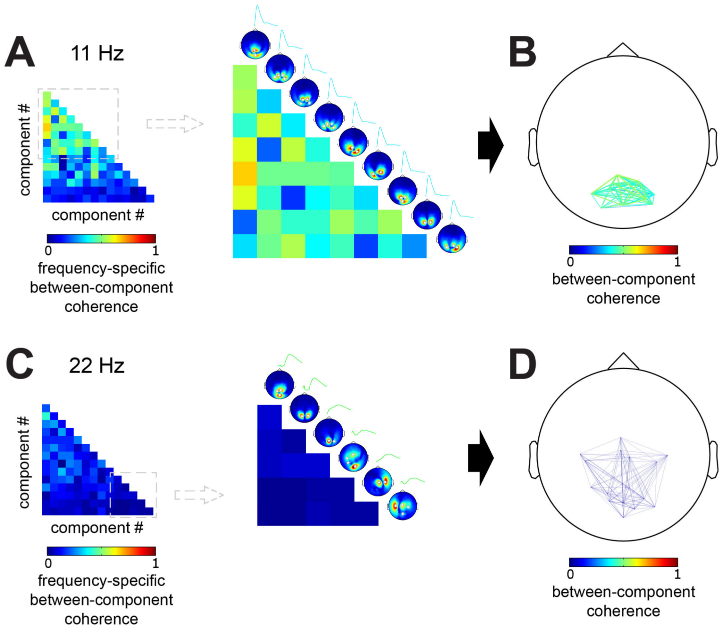

Figure B. **Weakly phase-coupled oscillatory networks (PCNs) are revealed by between-component coherence of the representative subject**. Weak PCNs reflect neuronal populations that are phase-coupled only in parts of a recording, and they can be identified by between-component coherence. Here we show weak PCNs formed by components of the representative subject. **A**, between-component coherence matrix for components reflecting alpha sources. Between-component coherence is calculated for each frequency, but only that of the peak frequency is shown (11 Hz). **B**, spatial distribution of alpha weak PCNs. Lines show peak-to-peak *between*-component connections (different from Figure A in S1 File panel B and C) and are drawn between peaks of different components. Line color indicates between-component coherence computed as the sum over frequencies, weighted by the product of the frequency profiles of the respective component-pair. Line thickness is the product of the amplitude at the peaks (spatial amplitude maps normalized to maximum of 1) and the coherence between both components. Peaks were detected by comparing the spatial amplitude map loadings between neighboring sensors (see

Supplementary Methods section 3), and are shown in A. **C,D** same as A,B but for beta components. Many alpha components were coherent and thus described weak PCNs. Interestingly, there was diversity in the between-component coherence: coherence between some components was weak, even though their coherence with other component was strong. Beta components did not have substantial between-component coherence and, as such, did not reveal any weak PCNs.

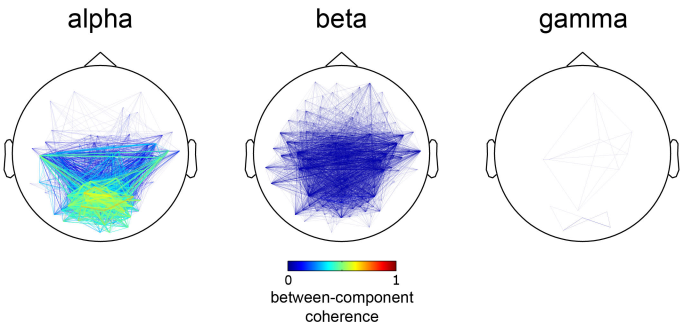

Figure C. **Weakly phase-coupled oscillatory networks (PCNs) are revealed by components from all datasets**. We show weak PCNs formed by components from all recordings. The spatial distribution of between-component coherence for alpha, beta and gamma components is shown. Peak-pair connectivity is constructed as before (Figure B in S1 File, panel B and D) but now components from all recordings are displayed together. Weak PCNs of alpha components were common, and were mostly posterior. Beta and gamma components did not show substantial between-component coherence, and as such did not describe weak PCNs.

Supplementary Discussion

Investigating PCNs using rhythmic components: strong alpha and beta networks and weak alpha networks

Our method estimates phase relations between interacting neuronal populations in two ways: within a component, and between components. These phase relations are the basis for identifying two types of PCNs (weak and strong), and allows for distinguishing them from single point sources. Distinguishing between PCNs and such point sources is important, because the sensor-level phase coupling produced by a point source reflects a single isolated population, whereas the sensor-level phase coupling of PCNs reflects communication between networks of neuronal populations. We additionally distinguish between strong and weak PCNs, which differ in the strength of the phase coupling between the underlying populations. We identified many alpha and beta strong PCNs, which most likely reflect interactions between distributed neuronal populations. Communication between distributed populations by alpha and beta phase coupling is in line with a possible role in top-down control and routing of information [1-3]. Phase coupling between distant areas has been reported before at beta [4-8], and at alpha [9-12] frequencies.

Our approach also allows us to identify weak PCNs. Whereas strong PCNs reflect highly consistent phase coupling between neuronal populations, weak PCNs are the result of weaker phase consistency between populations. Weak PCNs could reflect temporary networks: networks whose nodes (neuronal populations) interact for shorter periods of time (shorter than the duration of a trial). We only found alpha weak PCNs, and they mostly involved posterior sensors. This suggests a complex picture for the generation of posterior alpha, where nodes of the network may come and go. Temporary networks have been the topic of recent EEG/MEG studies [13-16], which show that these networks can form and dissolve at a time-scale as short as several hundred milliseconds. Interestingly, most of these studies find such networks in the beta band, whereas we only found alpha weak PCNs. This could be the result of different task demands, as these studies found either networks in resting state activity [13, 14, 16] or motor networks during a repetitive motor task [13]. In contrast, our tactile attention paradigm only had minimal motor demands. Alternatively, these different results could also be due to the fact that our networks are defined by phase coupling, whereas the other studies defined networks by correlations between band-limited amplitude envelopes.

Supplementary Methods

1. Between-component coherence can be accurately reconstructed in a two-step estimation procedure

To prevent components from splitting up into an arbitrary number of sub-components, we estimated the component-specific parameters under the constraint that the between-component coherency matrices are identity matrices. Then, in a second step, the between-component coherency matrices were estimated while keeping the component-specific parameters fixed. The identity matrix constraint unavoidably leads to some degradation of the component-specific parameters. As such, this two-step strategy is only valid under two assumptions: (1) the resulting component-specific parameter degradation is minimal, and (2) the between-component coherency matrices can be estimated reasonably well from these (degraded) parameters.

We evaluated the robustness of this two-step strategy in a simulation study in which we generated spatially overlapping MEG components with different levels of source-level coherence. From the simulated data, we extracted two components using the pipeline described above, and evaluated how well these components recovered the true parameter values (the component-specific parameters and the between-component coherency matrices). More specifically, we simulated two oscillatory neuronal sources with peak frequency at 10Hz and dipolar MEG sensor-level representations. We varied the sources' coherence by linearly mixing the source signals using three different degrees of mixing. This resulted in two source signals with a coherence of approximately 0.1, 0.5, and 0.9. The signals from the second source had a delay of 25ms relative to the first source (a quarter cycle at 10Hz). The two source signals were created from white noise to which the following operations were applied: (1) scaling of the amplitudes of its Fourier coefficients such that the amplitude spectrum was proportional to 1/f, giving the power spectrum a $1/f^2$ shape (Miller et al., 2009), and (2) band-pass filtering the resulting time domain signals between 8 and 12 Hz, using a 6th order Butterworth filter. The source signals were then projected to the sensor-level using lead fields that were obtained as follows. We started from a single-shell volume conduction model (Nolte, 2003), calculated from a T1-weighted MR image of the representative subject. Next, the two source locations and their source strengths in the x, y, z directions were chosen such that their lead fields were most similar to the spatial amplitude map and the spatial phase map of two components of the representative subject (see Results section 3; #6 and #8. Finally, these lead fields were scaled with trial profile loadings, as described in the following. The trial profile loadings were either 0.25, 0.50, or 0.75, and pairs of loadings (one loading for every component) were always non-identical. Each unique pair of coefficients was used equally often. To vary the signal-to-noise ratio (SNR), different amounts of spatially uncorrelated sensor-level noise (with a $1/f^2$ power spectrum) was added. However, as SNR did not substantially influence recovery, we only present the simulations with the lowest SNR (0.01). For each level of source mixing, we simulated 100 datasets, also denoted as *runs*. Each of these datasets consisted of 12 trials of 100s each.

The simulated datasets were analyzed in the same way as the real MEG recordings except that we only obtained CSDs for frequencies from 6 to 20Hz instead of from 6 to 40Hz. After extracting two components from each dataset, we investigated their recovery of the simulated parameters. The simulated spatial amplitude and spatial phase maps were obtained from the lead fields, and the simulated frequency profiles and between-component coherency matrices were obtained from a

spectral analysis of the source signals. This spectral analysis was identical to the one used for calculating the sensor-level CSDs. The simulated trial profiles are described above. The to-be-recovered parameters are shown in Figure D in S1 File, panel A. Recovery was quantified by coefficients that range from 0 to 1. These coefficients are described in Supplementary Methods section 2.

We show the recovery of the simulated spatial amplitude maps and spatial phase maps in Figure D in S1 File, panel B, the simulated frequency profiles in Figure D in S1 File, panel C, the simulated trial profiles in Figure D in S1 File, panel D, and the simulated between-component coherency in Figure D in S1 File, panel E. Recovery is shown as a function of source signal coherence. The main findings are the following: (1) spatial amplitude maps, spatial phase maps, frequency profiles, and trial profiles show near perfect recovery with low source signal coherence, (2) with increasing source signal coherence, recovery of the spatial phase maps and trial profiles diminishes slightly, (3) estimated between-component coherence increases with source signal coherence but systematically underestimates it, and (4) the average phase relation between the source signals are poorly recovered by the phase of between-component coherency.

In sum, we have shown that the component-specific parameters of phase-coupled components can be recovered well under the constraint of zero between-component coherence. Differences in source signal coherence are reflected in the estimated between-component coherence, although the source signal coherence itself is underestimated. The average phase relations between the source signals are estimated very poorly, and should therefore not be interpreted.

2. Coefficients for assessing the recovery of the simulated parameters

We calculated a number of coefficients to assess the recovery of the simulated parameters by the extracted components. We use three different coefficients: (1) one for the spatial amplitude maps, the frequency profiles, and the trial profiles, (2) one for the spatial phase maps, and, (3) one for the phase of the between-component coherence. Each coefficient had ranged from 0 to 1, with 1 reflecting perfect recovery. The first recovery coefficient was constructed as the component-specific inner product between the normalized extracted parameter vector (spatial amplitude map, frequency profile, trial profile) and its simulated counterpart. The recovery coefficient for the spatial phase maps, which is more complicated, is calculated as follows:

$$\frac{\sum_{k=1}^{K}\left[\left|sum\left[\left[\left(A^s \circ exp(i\lambda_k)\right)\cdot\left(A^s \circ exp(i\lambda_k)\right)^*\right] \circ \overline{\left[\left(A^s \circ exp(\imath\lambda_k^s)\right)\cdot\left(A^s \circ exp(\imath\lambda_k^s)\right)^*\right]}\right]\right| \cdot B_k^s\right]}{\sum_{k=1}^{K} B_k^s}$$

The calculation involves four steps. In the first step, we compute the frequency-specific between-sensor phase relations on the basis of the extracted phases $exp(i\lambda_k)$ and weight these by the normalized simulated spatial amplitude map, A^s. The results of this operation are stored in the square matrix $\left(A^s \circ exp(i\lambda_k)\right)\cdot\left(A^s \circ exp(i\lambda_k)\right)^*$, in which $exp(i\lambda_k)$ is a column vector, $\cdot$ denotes the matrix product, and $\circ$ the element-wise product. The weighting with the normalized simulated spatial amplitude map A^s ensures that the recovery coefficient is mostly determined by the sensors that are strongly affected by the simulated sources. In the second step, we calculate the simulated counterpart of the first matrix (using the simulated phases $exp(i\lambda_k^s)$), take its conjugate (denoted by the horizontal bar $\overline{\ }$), and perform an element-wise multiplication of the two matrices. This operation produces large values for sensor pairs whose extracted phase relation differs strongly from the simulated one. In the third step,

we summed these phase differences over all sensor-pairs, and take its absolute value, such that we obtain a frequency-specific recovery coefficient that has a range of 0 to 1. (To keep the formula simple, we define $sum[\quad]$ to be the sum over all sensor-pairs, which are organized in a matrix.) In the fourth step, we compute their weighted average over frequencies, with the weights obtained from the simulated frequency profile B^s. The resulting coefficient is sensitive to differences between the extracted and simulated spatial phase maps, and is determined most strongly by frequencies and sensors that are strongly affected by the simulated sources.

The recovery coefficient for the phase of between-component coherence was constructed as follows:

$$\left|\frac{\sum_{k=1}^{K}\left[\left(exp(i\varphi_k)\cdot\overline{exp(\imath\varphi_k^s)}\right)\cdot B_k^{s1}\cdot B_k^{s2}\right]}{\sum_{k=1}^{K}(B_k^{s1}\cdot B_k^{s2})}\right|$$

Computing this coefficient involves two steps. In the first step, we compute the frequency-specific phase difference between the phase of the extracted between-component coherency $exp(i\varphi_k)$ and the phase of its simulated counterpart $exp(i\varphi_k^s)$. In the second step, we compute the weighted average of these phase differences over frequencies, where the weights are obtained from the simulated frequency profiles of both components (B^{s1}, and B^{s2}). The resulting coefficient is sensitive to the phase of between-component coherency, with a weighting that ensures that it is mostly determined by frequencies that dominate the simulated sources.

3. Identifying peaks in spatial amplitude maps

To investigate phase-coupled oscillatory networks (PCNs) we identified peaks in the spatial amplitude maps. These peaks were identified in two steps. In the first step, we identified sensors that were a local maximum in the mini-map defined by this sensor plus its neighboring sensors, and which had an amplitude of at least 30% of the maximum within the whole map. In the second step, we pruned these local maxima such that sensors identified as peaks did not share neighboring sensors. In other words, no sensor identified as a peak shared a neighbor with another peak sensor. When peak sensors shared neighbors, only the peak sensor with the highest amplitude was kept. It has to be admitted that this procedure was not grounded in a biophysical rationale. Rather, it was chosen because it was both intuitively plausible and because it performed well in separating dipolar from non-dipolar spatial maps. Representative examples of detected peaks are shown in the Figure B in S1 File.

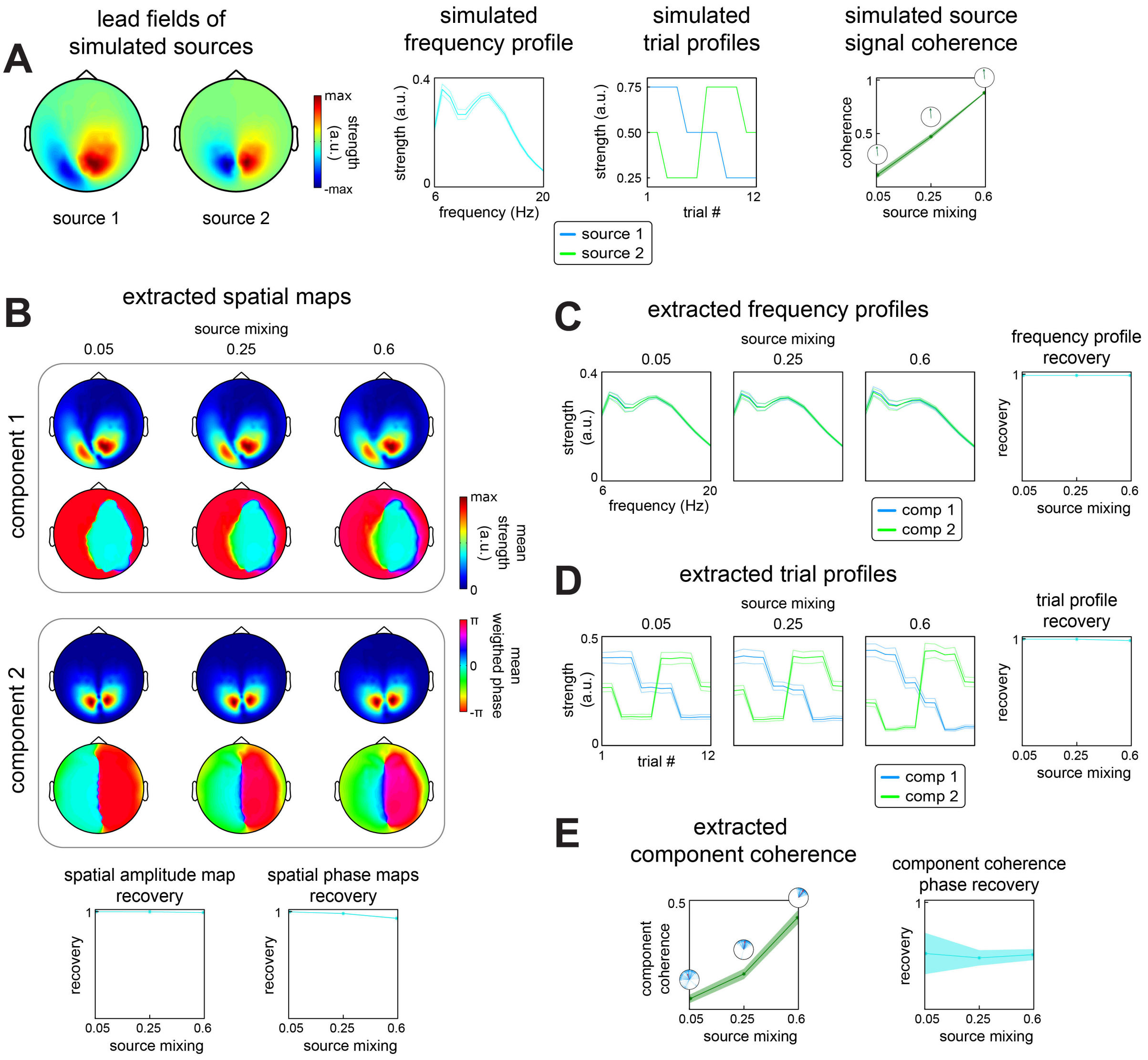

Figure D. **Simulations show phase-coupled components can be accurately extracted**. Weak PCNs can be formed by multiple phase-coupled components and are revealed by between-component coherence. However, components are extracted under a zero coherence constraint, and coherence is computed afterwards. This is to prevent a split-up of components into arbitrary numbers of subcomponents. This strategy is only valid if: (1) the resulting component-specific parameter degradation is minimal, and (2) between-component coherence can be estimated reasonably from these (degraded) parameters. Here we present simulations to test this strategy (for details see Supplementary Methods section 1 and 2). We simulated MEG recordings of two sources and systematically varied their coherence (source mixing of 0.05, 0.25, and 0.6). We generated 100 datasets per level of source mixing. Sensor-level measurements were generated by projecting the source signals through lead fields from the representative subject. Source signals were generated as band-passed noise with a 1/f^2 shaped power spectrum and consisted of 12 trials of 100s weighted by the trial profile. Spatially uncorrelated noise with a 1/f^2 shaped power spectrum was added after projecting source signals to the sensor-level. We only show results for the worst signal-to-noise ratio (0.01). Components were extracted from each of 3x100 datasets using the same analysis pipeline as for the real MEG recordings, and we evaluated whether these components accurately recovered the simulated sources. **A**, source lead fields, frequency profiles, trial profiles, and source signal coherence. Frequency profiles shown are the average of the two simulated components, averaged over runs. Source signal coherence shown is the average over runs of the sum of coherence over frequencies, weighted by the product of both frequency profiles. The phase of coherence shown in polar plots was constructed as the mean resultant vector over runs, of the run-specific average phase over frequencies, which was weighted by the product

of both frequency profiles. Thin lines in the frequency profiles and shaded area of the source signal coherences reflects the SD. **B**, recovery of the simulated spatial amplitude map and spatial phase maps (constructed from the lead fields). Spatial amplitude maps shown are averaged over runs. Spatial phase maps shown are also averaged over runs, weighted by the simulated frequency profiles. Recovery coefficients reflect average recovery accuracy over runs (shaded areas reflects SD), averaged over both components and range from 0 to 1 (perfect recovery). **C**, same as B but for the frequency profiles. Thick lines display the average frequency profiles, thin lines the SD. **D**, identical to C but for the trial profiles. **E**, recovery of source coherence by between-component coherence. Between-component coherence shown is averaged over runs (shaded area reflects SD), and was constructed as the sum over frequencies per run, weighted by the product of the simulated frequency profiles. Polar plots show the run-specific average phase, weighted in the same way (red arrow reflects mean resultant vector over runs). The results show that the spatial amplitude maps, spatial phase maps, frequency profiles, and trial profiles are minimally impacted by the zero coherence constraint. Between-component coherence was estimated reasonably well, but its phase relations were not. As such, our analysis strategy is valid as long as phase relations between components are not interpreted.

References

1. Palva S, Palva JM. New vistas for alpha-frequency band oscillations. Trends Neurosci. 2007;30(4):150-8.
2. Jensen O, Gips B, Bergmann TO, Bonnefond M. Temporal coding organized by coupled alpha and gamma oscillations prioritize visual processing. Trends Neurosci. 2014;37(7):357-69.
3. Engel AK, Fries P. Beta-band oscillations - signalling the status quo? Curr Opin Neurobiol. 2010;20(2):156-65.
4. Buschman TJ, Miller EK. Top-down versus bottom-up control of attention in the prefrontal and posterior parietal cortices. Science. 2007;315(5820):1860-2.
5. Brovelli A, Ding MZ, Ledberg A, Chen YH, Nakamura R, Bressler SL. Beta oscillations in a large-scale sensorimotor cortical network: Directional influences revealed by Granger causality. Proc Natl Acad Sci U S A. 2004;101(26):9849-54.
6. Pesaran B, Nelson MJ, Andersen RA. Free choice activates a decision circuit between frontal and parietal cortex. Nature. 2008;453(7193):406-U61.
7. Canolty RT, Ganguly K, Kennerley SW, Cadieu CF, Koepsell K, Wallis JD, et al. Oscillatory phase coupling coordinates anatomically dispersed functional cell assemblies. Proc Natl Acad Sci U S A. 2010;107(40):17356-61.
8. Gross J, Schmitz F, Schnitzler I, Kessler K, Shapiro K, Hommel B, et al. Modulation of long-range neural synchrony reflects temporal limitations of visual attention in humans. Proc Natl Acad Sci U S A. 2004;101(35):13050-5.
9. Halgren E, Boujon C, Clarke J, Wang C. Rapid distributed fronto-parieto-occipital processing stages during working memory in humans. Cereb Cortex. 2002;12(7):710-28.
10. Palva JM, Palva S, Kaila K. Phase synchrony among neuronal oscillations in the human cortex. J Neurosci. 2005;25(15):3962-72.
11. Pollok B, Gross J, Muller K, Aschersleben G, Schnitzler A. The cerebral oscillatory network associated with auditorily paced finger movements. Neuroimage. 2005;24(3):646-55.
12. von Stein A, Chiang C, Konig P. Top-down processing mediated by interareal synchronization. Proc Natl Acad Sci U S A. 2000;97(26):14748-53.
13. Woolrich MW, Baker A, Luckhoo H, Mohseni H, Barnes G, Brookes M, et al. Dynamic state allocation for MEG source reconstruction. Neuroimage. 2013;77:77-92.
14. de Pasquale F, Della Penna S, Snyder AZ, Lewis C, Mantini D, Marzetti L, et al. Temporal dynamics of spontaneous MEG activity in brain networks. Proc Natl Acad Sci U S A. 2010;107(13):6040-5.
15. Breakspear M, Williams LM, Stam CJ. A novel method for the topographic analysis of neural activity reveals formation and dissolution of 'dynamic cell assemblies'. J Comput Neurosci. 2004;16(1):49-68.
16. Brookes MJ, O'Neill GC, Hall EL, Woolrich MW, Baker A, Corner SP, et al. Measuring temporal, spectral and spatial changes in electrophysiological brain network connectivity. Neuroimage. 2014;91:282-99.
17. Miller KJ, Sorensen LB, Ojemann JG, den Nijs M. Power-Law Scaling in the Brain Surface Electric Potential. PLoS Comput Biol. 2009;5(12).
18. Nolte G. The magnetic lead field theorem in the quasi-static approximation and its use for magnetoencephalography forward calculation in realistic volume conductors. Phys Med Biol. 2003;48(22):3637-52.
